# Valproic acid-induced teratogenicity is driven by senescence and prevented by Rapamycin in human spinal cord and animal models

**DOI:** 10.1038/s41380-024-02732-0

**Published:** 2024-09-03

**Authors:** Giovanni Pietrogrande, Mohammed R. Shaker, Sarah J. Stednitz, Farhad Soheilmoghaddam, Julio Aguado, Sean D. Morrison, Samuel Zambrano, Tahmina Tabassum, Ibrahim Javed, Justin Cooper-White, Thomas P. Davis, Terence J. O’Brien, Ethan K. Scott, Ernst J. Wolvetang

**Affiliations:** 1 Australian Institute for Bioengineering and Nanotechnology, The University of Queensland, St. Lucia, Brisbane, QLD 4072 Australia; 2 Department of Anatomy & Physiology, University of Melbourne, Parkville, VIC Australia; 3 School of Chemical Engineering, University of Queensland, St. Lucia, QLD 4072 Australia; 4 School of Medicine, Vita-Salute San Raffaele University, Milan, 20132 Italy; 5 Division of Genetics and Cell Biology, San Raffaele Scientific Institute, Milan, 20132 Italy; 6 Department of Neuroscience, The Central Clinical School, Alfred Health, Monash University, Melbourne, VIC Australia; 7 The Departments of Medicine and Neurology, The Royal Melbourne Hospital, The University of Melbourne, Parkville, VIC Australia; 8 Queensland Brain Institute, The University of Queensland, St. Lucia, Brisbane, QLD 4072 Australia

**Keywords:** Neuroscience, Drug discovery, Stem cells, Biochemistry

## Abstract

Valproic acid (VPA) is an effective and widely used anti-seizure medication but is teratogenic when used during pregnancy, affecting brain and spinal cord development for reasons that remain largely unclear. Here we designed a genetic recombinase-based *SOX10* reporter system in human pluripotent stem cells that enables tracking and lineage tracing of Neural Crest cells (NCCs) in a human organoid model of the developing neural tube. We found that VPA induces extensive cellular senescence and promotes mesenchymal differentiation of human NCCs. We next show that the clinically approved drug Rapamycin inhibits senescence and restores aberrant NCC differentiation trajectory after VPA exposure in human organoids and in developing zebrafish, highlighting the therapeutic promise of this approach. Finally, we identify the pioneer factor AP1 as a key element of this process. Collectively our data reveal cellular senescence as a central driver of VPA-associated neurodevelopmental teratogenicity and identifies a new pharmacological strategy for prevention. These results exemplify the power of genetically modified human stem cell-derived organoid models for drug discovery.

## Introduction

Valproic acid (VPA) is widely prescribed for the treatment of bipolar disorder, migraine, and epilepsy [1]. It is the most effective anti-seizure medication (ASM) for the treatment of generalised epilepsy syndromes [2, 3] and influences voltage-dependent sodium channels, GABA neurotransmitter release, HDAC-mediated epigenetic modifications, modulation of neurotropic factor production, and dendritic spines re-organization [4]. However, its use during pregnancy leads to an increased risk of teratogenesis termed Foetal Valproate Syndrome (FVS, OMIM #609442). FVS encompasses heart defects, severe craniofacial defects, neural tube defects, and neurocognitive disorders. Consequently, VPA is considered contraindicated for use during pregnancy in many countries [5], and prescription to women of childbearing potential is not recommended even in absence of plans for pregnancy. Regrettably, numerous patients lack alternative therapeutic options with equivalent therapeutic effectiveness [6, 7] making more stringent guidelines like the ones recently adopted in the United Kingdom exceptionally controversial [8]. Studies in zebrafish, mice, rats, chickens, and human cellular models have shown similar teratogenic effects of VPA [9–13], suggesting a largely evolutionary conserved fundamental pathogenic process. Strikingly, Neural Crest cells (NCCs) are involved in the development of most embryonic tissues affected by VPA.

During development NCCs migrate from the neural tube and differentiate into diverse cell types, including melanocytes, peripheral neurons, smooth muscle cells and mesenchymal stem cells (MSCs) [14], contributing to the formation of multiple tissues. Remarkably few reports have investigated the impact of VPA exposure on NCC, and these were limited to in vitro cultured cells [15], or investigated palate structure [16]. Thus, it remains unclear whether VPA causes anomalous behaviour of NCC and if this is linked to developmental defects of the nervous system, as observed in newborns.

To elucidate the mechanism of VPA-induced teratogenicity, we engineered a *SOX10:mMaple-fLPo/eGFP* human induced pluripotent stem cell (hiPSC) line that enables a genetic switch that facilitates detection and lineage tracing of SOX10^+^ NCC across multiple lineages. We next use this approach to detect variations in physiologically relevant behaviour of NCC in a 3D human neural tube derived spinal cord organoid model (hSCOs). We found that VPA exposure promotes inappropriate differentiation of newly specified NCCs into MSC at the expense of neuronal lineages and revealed a strong connection with the widespread rise in cellular senescence. Indeed, the senomorphic drug Rapamycin [17] prevents the increase in VPA-induced senescence and associated NCC differentiation preventing motor-sensory neuron specification. Remarkably, in vivo zebrafish model replicates the VPA-induced NCC differentiation defects and the subsequent Rapamycin rescue, providing additional validation of the biological significance of our findings beyond the in vitro setting. Finally, we identify the master transcription factor AP1 as a likely mediator of VPA-induced senescence and aberrant differentiation. Collectively our data shed light on the delicate balance between cellular senescence and cell fate choices that underlie the teratogenicity of VPA and identifies a potential pharmacological prevention strategy through Rapamycin treatment.

## Results

### Conditional genetic switch for *SOX10* gene expression report and lineage tracing

**Fig. 1:**
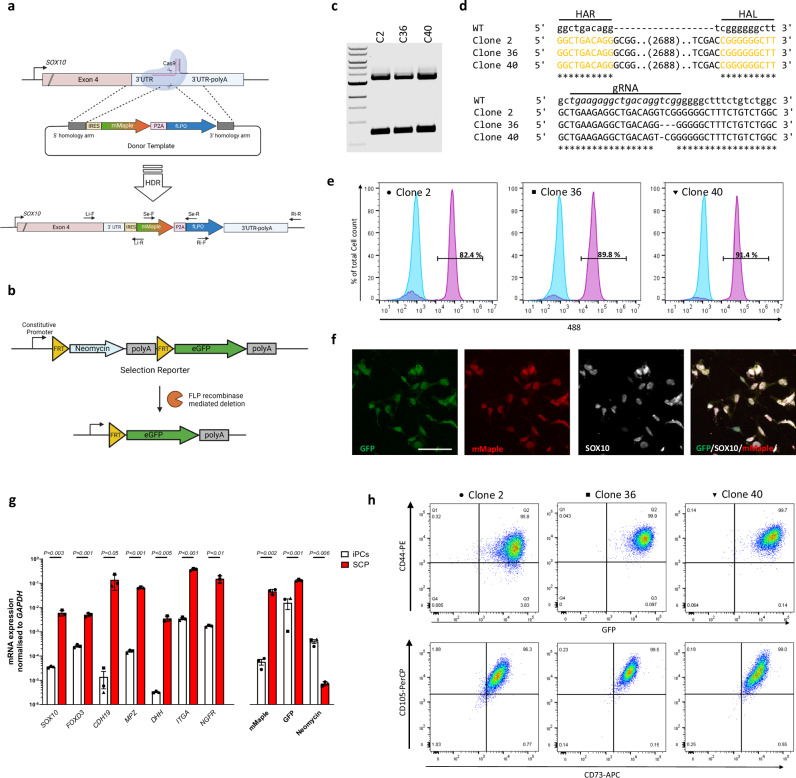
Generation of specific and lasting genetic switch driven by *SOX10.* **a** Schematic representation of the 3’ UTR region of the *SOX10* locus showing the CRISP-Cas9 targeted area and donor template mediated strategy for insertion of the (mMaple)/recombinase (FLPo) cassette with an Internal Ribosome Entry Site (IRES) sequence within the UTR region. The indicated primer pairs are used in the following characterizations: Li-F/R left arm recombination, Ri-F/R right arm recombination, Se-F/R genetic switch expression. **b** Schematic of the Neomycin selection reporter cassette and activated state expressing GFP after FLPo mediated recombination. **c** Three randomly selected clones were screened for genetic switch insertion within the targeted locus by amplification with the Li-F/Ri-R primer pair. The higher band of 3.5 kb is consistent with monoallelic integration, while the band of 700 bp is expected by WT locus. 1Kb ladder. **d** Sanger sequencing of the upper and lower bands from (**c**) to verify homologous recombination of the homology arm or the presence of indels. gRNA sequence in italic. **e** Percentage of 488 positive cells assessed by FACS of each iPSC clone before (blue) and after (purple) SCP differentiation protocol. **f** Representative image showing SOX10 immunostaining and colocalization with GFP and mMaple in 2D differentiated SCPs. Scale bar 10 µm. **g** Relative mRNA abundance of the indicated markers and genetic switch element normalized to *GAPDH* mRNA in SCPs and iPSC clones. Each dot represents one clone. Error bars represent s.e.m.; two-tailed Student’s t-test. **h** FACS analysis showing emission in the 488 channel and expression of a panel of mature MSCs markers CD44, CD73 and CD105 in MSCs derived from SCP cells described in (e). Isotypic control incubation on primary hMSCs was used to set negative gating.

We designed a *SOX10*-driven genetic switch that upon activation leads to the expression of a *SOX10*-driven reporter and constitutive expression of a second reporter for permanent lineage tracing of the progeny derived from *SOX10* expressing cells. We used Cas9-mediated gene targeting to edit the 3’ UTR region of *SOX10*, 125 bp after the stop codon to insert an IRES-mMaple-P2A-fLPo expression cassette (Fig. 1a and SFig. 1a). This first element is directly dependent on *SOX10* transcription and leads to the concomitant expression of a fluorescent reporter (mMaple [18]) and the DNA recombinase Flp [19]. The second element (Fig. 1b) was randomly integrated into the genome and drives constitutive expression of a Neomycin-selectable module that, after excision by Flp [20], enables constitutive GFP-expression based lineage tracing. We initially verified the functionality of this switch by differentiating the targeted bulk hiPSC population into Schwann Cells Precursors (SCPs), a *SOX10* expressing cell type. mMaple expression was functionally confirmed by FACS (SFig. 1b) and photoconversion (SFig. 1c).

### *SOX10* genetic switch is only activated upon differentiation into *SOX10* expressing cells and preserved across multiple cell types

Starting from the bulk population of edited iPSCs containing the genetic switch we isolated Clone 2, Clone 36, and Clone 40 with correct integration of the *mMaple/fLPo* cassette (Fig. 1c, d). Clone 36 and Clone 40 presented indels on the other *SOX10* allele, generated during the Cas9 gene editing process (Fig. 1d). We again functionally evaluated the efficiency of the switch in each clone by differentiation into SCPs. The genetic switch was specifically activated in SOX10 positive cells only (Fig. 1e, f and SFig. 1d). Importantly, neither the original iPSC bulk population nor any of the iPSC clones presented a detectable amount of 488 fluorescence positive cells (Fig. 1e, blue histogram and SFig. 1b), highlighting the strong specificity of our system that is only activated by substantial expression of endogenous *SOX10*. Robust differentiation is shown by the sharp decrease of pluripotency markers (SFig. 1e) and upregulation of a panel of SCPs markers and genetic switch elements (Fig. 1g), while antibiotic resistance fades as consequence of the Flp recombinase-driven excision of the Neo expression cassette. This result was further supported by the selective death of GFP positive SCPs induced in the presence of G418 (SFig. 1f). To validate the robustness of lineage tracing across multiple lineages we differentiated SCPs from each of the three clones, sorted the GFP positive population and further differentiated the resulting population into MSCs. We found that more than 99% cells were positive for MSC markers, lost mMaple expression, and retained GFP expression for at least 12 weeks and after further differentiation into Osteoblasts and Adipocytes [9] (Fig. 1h and SFig. 2).

### Neural crest cells originate and differentiate in a 3D model of neural tube development

**Fig. 2:**
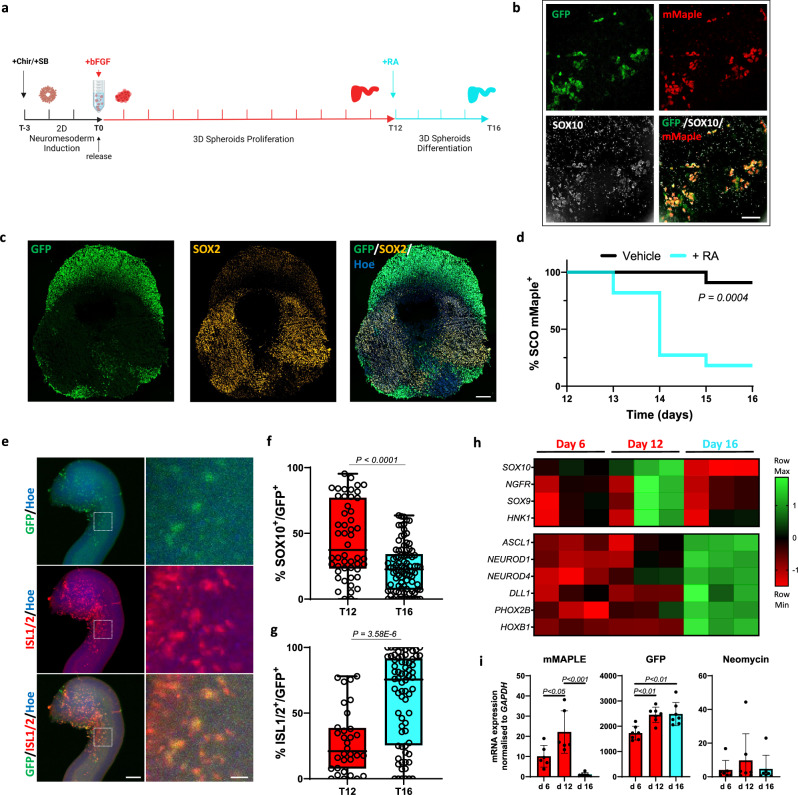
*SOX10* driven genetic switch is activated in SCOs and enables lineage tracing. **a** Schematic representation of the SCO differentiation protocol. Stages are divided into 2D/neuromesodermal induction (black), 3D proliferation/specification (red) and 3D differentiation (light blue). **b** Representative field of photoconverted d12 SCO immunolabelled whole mount for SOX10 (white) and overlay with eGFP (green) and mMaple (red). Scale bar 50 µm. **c** Representative image of sagittal section of an elongating SCO at day 12 immunolabelled for SOX2 (orange) and overlay with GFP (488 nm, green). Scale bar 100 µm. **d** Kaplan–Meier curve of mMaple positive SCOs. Organoids were imaged every day from day 12 to 16 during exposure to retinoic acid (RA) (n = 11) or vehicle (n = 11) and evaluated for the presence of photoconverted (mMaple positive) cells within the organoid. Long-rank test. **e** Representative field of whole mount d16 SCO immunolabelled for ISL1/2 (red) and overlay with eGFP (green). Left scale bar 70 µm, right scale bar 35 µm. **f** Box plots show the percentage of SOX10 and (**g**) ISL1/2 positive cells relative to the total number of GFP positive cells. Each data point in the box plot represents an ROI of an organoid section where GFP cells were present. Whiskers represent min-max values; d12 n = 33, d16 n = 67 ROI total were analysed; two-tailed Student’s t test. **h** Total RNA from 5 to 7 pooled SCOs was extracted at different stages of differentiation and used to quantify the mRNA expression levels of the indicated neural crest cells and neuronal markers and were normalizated to *GAPDH* transcript. The significance threshold was set to P = 0.01 as determined by one-way ANOVA with Tukey’s multiple-comparison post hoc corrections. Each column represents one of three independent differentiation batches sampled at the indicated timepoint. **i** Total RNA from 3 to 5 pooled SCOs of 6 (mMaple) and 7 replicates was extracted at different stages of differentiation and used to quantify the mRNA expression levels of the indicated genetic switch element normalized to *GAPDH* mRNA. Error bars represent s.d.; two-tailed Student’s t test.

3D organoid models can recapitulate developmental signalling cascades more closely than a 2D system, and are an excellent platform to study human development, drug toxicity or teratogenicity [21]. We adapted a recently described protocol to generate neural tube derived spinal cord organoid (SCO) [22] from human iPCS (Fig. 2A). Wholemount immunostaining for BRN2 showed the establishment of an elongating neuroepithelial layer after neuromesodermal induction and following 4 days of bFGF (SFig. 4a). We next observed the appearance of GFP/mMaple/SOX10 positive cells after only 6 days of bFGF, culminating at day 12 (SFig. 4b). Wholemount immunostaining confirmed that GFP/mMaple positive cells (Sfig. 3a, b) express SOX10 (Fig. 2b). Interestingly, SOX2 expressing progenitor cells and GFP expressing neural crest showed a distinct pattern within the elongating tube (Fig. 2c). Interruption of FGF, and exposure to retinoic acid (RA) for 4 days promoted loss of SOX10/mMaple expression (Fig. 2d) and SOX10+ cell differentiation towards ISL1/2^+^ neurons (Fig. 2e–g), mirroring events of in vivo ventral neural tube development [23], as previously reported [22]. We confirmed our observations in three independent organoid batches by qPCR analysis which showed increased expression of multiple trunk NCC markers [24] (Fig. 2h) and genetic switch elements (Fig. 2i) over time, that were turned off after RA addition in favour of markers of neuronal lineage. Next, we leveraged mMaple and GFP expression to study the behaviour of NCC within SCOs in real time (Video 1). Over 6 days we observed both de novo specification (Sfig. 3c) and proliferation (Sfig. 3d) of mMaple+ NCCs. Using AI-based segmentation [25] and tracking software [26] we tracked GFP positive cells and identified two groups of mMaple positive and negative cells that locate in different regions (Sfig. 3e). Finally, Sox10:mMaple positive cells display higher motility than differentiated crest-derived cells (Sfig. 3f and g and Movie 2), consistent with in vivo observations [27]. We conclude that our lineage tracing approach together with live imaging enables high throughput and scalable screening and allows the identification of compounds that can perturb the behaviour and/or cell identity of cells of interest within human organoid models.

### Valproic acid (VPA) causes premature differentiation and SASP (senescence associated secretory phenotype) induced cellular senescence

**Fig. 3:**
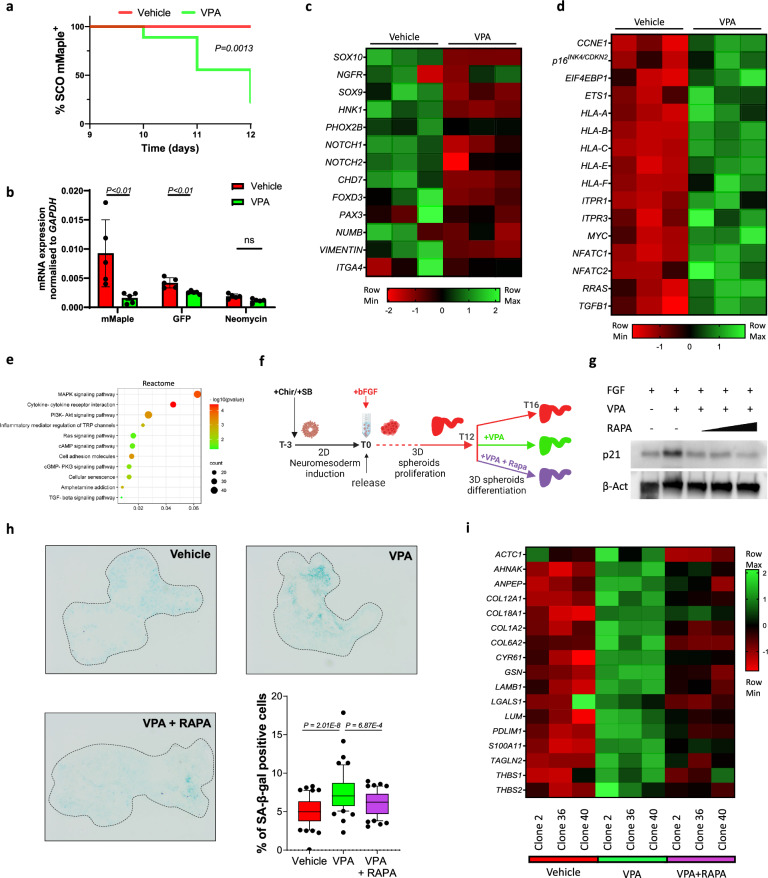
VPA induces NCC differentiation and promotes cellular senescence. **a** Kaplan–Meier curve of mMaple positive SCOs. Organoids were imaged every day from day 9 to 12 during exposure to vehicle (n = 10) or VPA (n = 9) and evaluated for the presence of photoconverted (mMaple positive) cells within the organoid. Long rank test. **b** Total RNA from 3-5 pooled SCOs of 5 replicates exposed to vehicle or VPA was extracted at different stages of differentiation and used to quantify the mRNA expression levels of the indicated genetic switch element normalized to *GAPDH* mRNA. Error bars represent s.d.; two-tailed Student’s t test. **c** 5-7 SCOs generated from each clone were pooled, total RNA was extracted after exposure to vehicle or VPA and used for bulk RNA sequencing to quantify the expression levels of the indicated neural crest cells markers and **d** senescence markers. Each column in the heatmap represents one of three clones. **e** Gene Set Enrichment Analysis was carried out using DAVID. The statistically significant senescence signatures were selected (FDR < 0.25) and placed in order of enrichment. Each pathway is enriched in VPA treatments compared to vehicle-treated organoids. **f** Schematic representation of experimental design of SCOs exposure to Rapamycin. **g** Western blot analysis of organoids exposed to FGF alone (vehicle), VPA and VPA and increasing concentrations of Rapamycin, 125, 250 and 500 nM left to right. **h** SA-β-gal assays were performed on organoid sections. Each data point in the whisker box plot represents a single section analysed. Error bars represent s.d.; at least 12 individual organoids were analysed per condition; SDs significantly different (F30.23 P < 0.0001), one-way Brown-Forsythe and Welch ANOVA, the significance of each comparison is indicated in the graph. **i** Heatmap showing Senescence-Associated Secretory Phenotype (SASPs) expression generated from bulk RNA sequencing of organoids exposed to vehicle, VPA and VPA + Rapamycin identified on SASP atlas as per **c**.

**Fig. 4:**
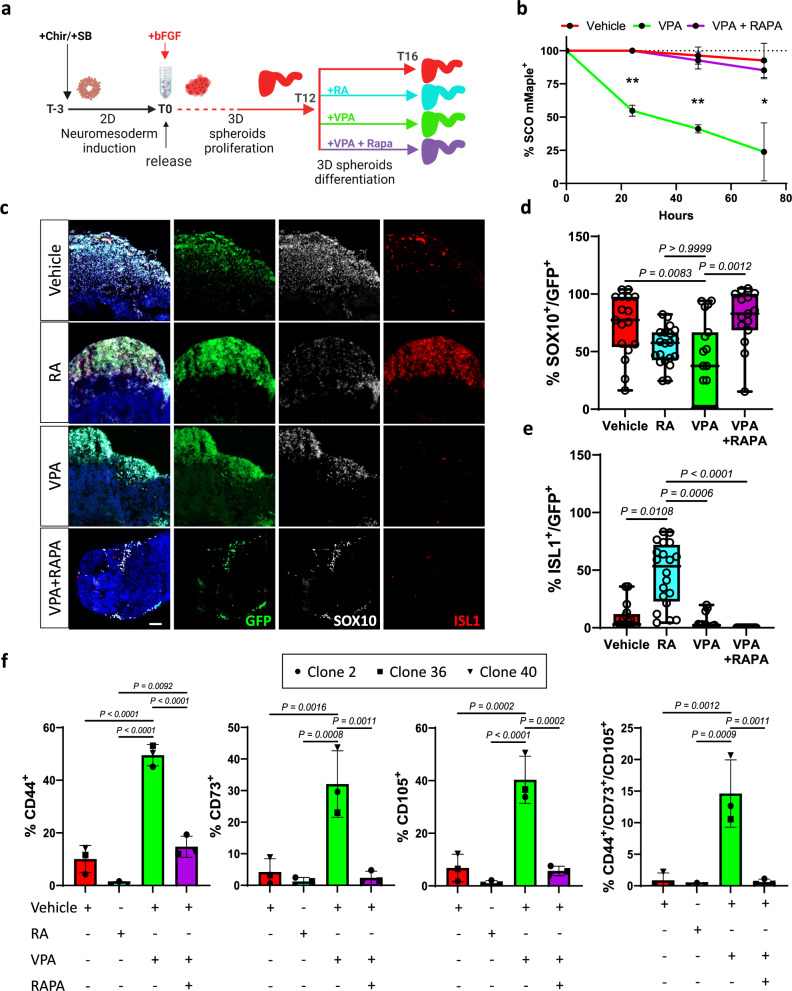
VPA promotes NCC differentiation into Mesenchymal Stem cells. **a** Schematic representation showing the timeline of organoids exposure to the indicated compounds and combinations. **b** Graph showing the percentage of mMaple positive SCOs over time during exposure to the indicated compounds. Organoids were imaged every day from day 12 to 16 during exposure to vehicle (C2 n = 7, C36 n = 6, C40 n = 6), VPA (C2 n = 7, C36 n = 7, C40 n = 8), or VPA+Rapamycin (C2 n = 9, C36 n = 9, C40 n = 9), and evaluated for the presence of photoconverted (mMaple positive) cells within the organoid. Two-way ANOVA analysis attributed a significant variation to treatment (53,42%, p = 0.0001) and time (20,75%, p = 0.0004). The significance of VPA to other groups per each timepoint is indicated on the graph, *p < 0.05, **p < 0.005. **c** Representative fields of sections of organoids after multiple treatments immunolabelled for SOX10 (white) and ISL1 (red). Scale bar 50 µm. **d** Quantification of the colocalization of SOX10 with GFP and **e** ISL1 with GFP presented in **c**. Each point of the box plot represents an ROI on a section. 8-12 organoids generated from each clone and pooled were analyzed; one-way ANOVA Kruskal-Wallis with multiple comparison post hoc correction. **f** Quantification by FACS analysis of GFP+ cells presenting mesenchymal stem cells markers and quantification of triple positive % in organoids generated from each clone after exposure to multiple compounds. RAPA refers to VPA+Rapamycin. Error bars represent s.d.; One-way ANOVA with multiple comparison post hoc correction.

After establishing that we developed a system that reliably reports on the presence and differentiation of NCCs, we investigated the aetiology of neural tube malformations caused by VPA towards the end of the proliferation phase (T9 to T12) before maturation (T12-T16). Isolated mMaple+ organoids were exposed for 72 h to FGF and 2 mM VPA (the highest dosage with clinical relevance [28], subsequently referred to as VPA unless otherwise specified). After exposure, 100% of the FGF group maintained the presence of mMaple+ NCC as expected (SFig. 4b), while 82% of the organoids in VPA group did not show detectable mMaple signal (Fig. 3a and SFig. 4c). This decrease in SOX10:mMaple expression was confirmed at transcriptional level (Fig. 3b) and was associated with a decrease in the expression of NCC markers (SFig. 4d) that was remarkably consistent at lower VPA concentrations (Fig. 4e). Bulk RNA sequencing performed on SCOs generated from the three previously described clones C2, C36 and C40 treated with VPA revealed downregulation of a panel of NCC associated transcripts (Fig. 3c). Intriguingly, *SOX10* was the second most significantly deregulated gene following VPA exposure (*Padj* = 3.59E–39). Further analysis of the bulk RNA seq revealed a substantial upregulation of a large pool of senescence associated genes [29] (Fig. 3d) and enrichment for KEGG pathways closely associated with proliferation, autophagy and inflammation (Fig. 3e) like MAPK and AKT pathways, an observation corroborated by the substantial increase in phospho-ERK and phospho-AKT levels (SFig. 4f) [30]. Interestingly, VPA-induced expression of senescence-associated genes [31] is also triggered in a different organoid model of the initial phases of neural tube folding [32] (Stable 2). To test the biological significance of the observed increase in senescence markers we next exposed SCOs generated from three different clones to VPA and VPA + Rapamycin, an efficient suppressor of cellular senescence [33] (Fig. 3f). We found that Rapamycin prevented the VPA-induced upregulation of senescence marker p21^CIP1/WAF^ (Fig. 3g) and the increased activity of senescence associated (SA)-β-Galactosidase brought about by VPA (Fig. 3h). Strikingly, Rapamycin affected the expression of Senescence-associated secretory phenotype (SASP) [34] induced by VPA (Fig. 3i) as well as other hallmarks of cellular senescence like proliferation and DNA damage repair [35] (SFig. 4g). These results suggest that dysregulated SASP expression and induction of cellular senescence during development can contribute to the teratogenic properties of VPA and are prevented by Rapamycin.

### Rapamycin prevents precocious differentiation of NCCs towards mesenchymal lineage caused by VPA

After we identified senescence as a potential driver of NCCs differentiation, we aimed to dissect NCC fate choices, and test whether Rapamycin could prevent this process (Fig. 4a). We observed that VPA causes precocious NCC differentiation in SCOs generated from all three clonal lines, as indicated by the loss of mMaple positive cells. Importantly, this was significantly prevented by Rapamycin (Fig. 4b) while the total number of GFP+ cells was unchanged (SFig. 4h). We next leveraged our lineage tracing to unravel the identify the progeny derived from NCCs (Fig. 4c). As expected, in vehicle SCOs most of GFP+ positive cells express SOX10 (Fig. 4d), while after exposure to RA these SOX10+ cells switch towards the neuronal lineage (Fig. 4e). VPA treatment substantially decreased the presence of SOX10+ cells but did not lead to an increased presence of neuronal cells (Fig. 4e and SFig. 4i). Indeed, genes significantly repressed (*Padj* < 0.05) after VPA exposure are mostly expressed in neurons, as indicated by their mapping to the neuronal cluster (SFig. 4j) of single cell transcriptomic atlas of human spinal cord [36]. Remarkably, Rapamycin preserved the SOX10+ population, and thus prevented the aberrant differentiation of NCCs induced by VPA exposure. Transcriptional data from bulk (Sfig. 5a) and clonal iPSC lines (Sfig. 5b) derived organoids treated with VPA revealed an increased expression of MSC markers, and genes significantly induced by VPA exposure (*Padj* < 0.05) map to a foetal mesenchymal progenitors’ cluster (Sfig. 5c) identified by scRNA sequencing of human foetal spinal cord [37], further suggesting VPA exposure results promotes NCC specification into MSC. However, these gene expression changes were not observed after exposure to RA (Sfig. 5d). Rather, VPA exposure impaired the specification of NCCs-derived neuronal cells (Sfig. 5e). In support of these data, FACS analysis revealed that upon VPA exposure a substantial portion of GFP+ cells present the canonical MSCs markers CD44, CD73 and CD105 (Fig. 4f and SFig. 6) and when isolated, they proliferate, and survive in MSC medium (SFig. 5f, g), while GFP negative cells display only a negligible (<0.5%) level of these markers (Sfig. 7). Intriguingly, data show that Rapamycin prevents this process. Thus, we conclude that VPA exposure biases NCC differentiation into MSCs at the expense of neuronal differentiation, and addition of Rapamycin is sufficient to restore this specification.

### Rapamycin prevents precocious differentiation of NCCs caused by VPA in developing zebrafish

**Fig. 5:**
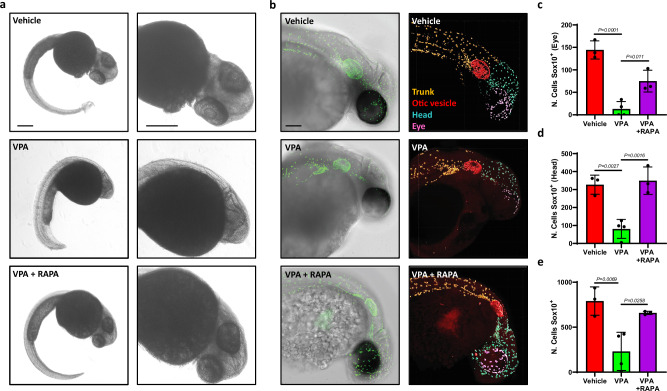
Rapamycin prevents VPA-induced promotion of NCC differentiation in Zebrafish. **a** Representative image of zebrafish larvae and zoom of the cranial region at 48 hpf exposed to the indicated compounds since 2 hpf. **b** Representative images of bright field overlayed with confocal imaging of zebrafish larvae exposed to multiple compounds and immunolabeled for Sox10 (green) and 3D reconstruction of identified Sox10+ cells within the larvae. The upper body was partitioned into multiple regions, with each Sox10+ cell being assigned a specific colour corresponding to its area. Scale bar 100 µm. **c** Quantification of the number of Sox10 positive cells counted in the palate region (purple), **d** head, and **e** entire upper body of zebrafish larvae exposed to vehicle, VPA and VPA+Rapamycin. Each point of the bar graph represents one larva. Error bars represent s.d.; one-way ANOVA with multiple comparison post hoc correction.

To confirm that Rapamycin treatment can prevent VPA induced differentiation of NCCs in developing animals, we exposed fertilised zebrafish eggs to vehicle, VPA and VPA + Rapamycin from fertilization until hpf 48. We noticed that most of the controls broke through the embryo chorion while VPA treated embryos often failed to achieve this (results not shown). Defects in the development of embryos exposed to VPA were macroscopic and severely affected the development of the craniofacial area, dorsal orbit and the eye (Fig. 5a) [38]. Whole mount immunostaining for sox10 (Fig. 5b) uncovered a significant decrease in sox10+ cells in all regions analysed (Fig. 5e), including the areas of palate (Fig. 5c) and neck/head (Fig. 5a). Interestingly, Rapamycin exposure was sufficient to prevent the loss of sox10+ cells induced by VPA, further supporting our working hypothesis that VPA alters the behaviour of NCC and their progeny and this can be prevented by Rapamycin.

### AP1 activity is necessary for VPA induced defects

**Fig. 6:**
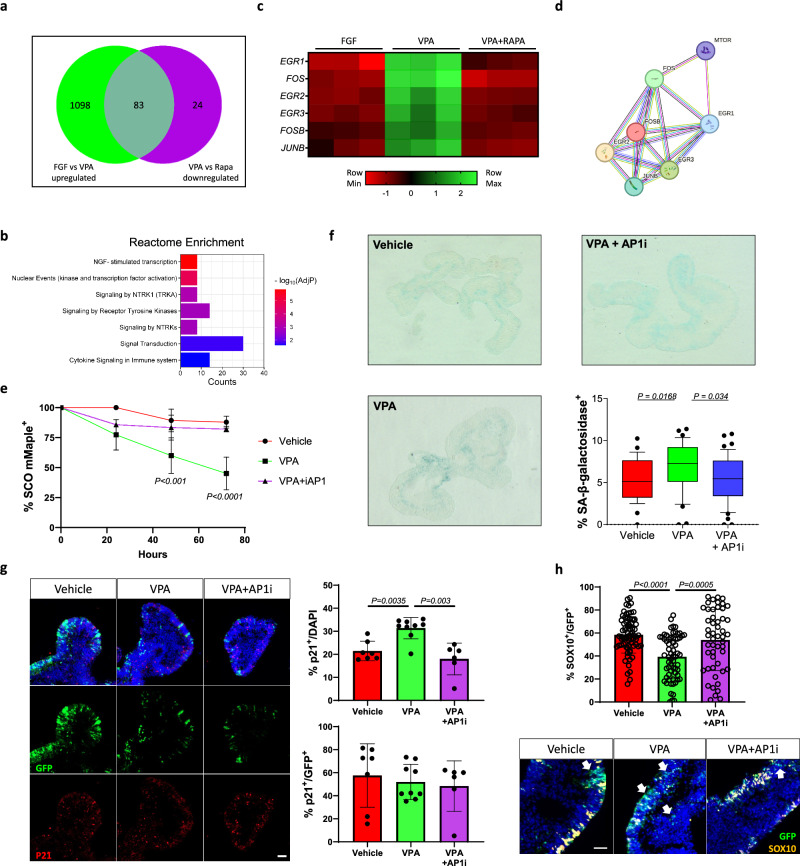
VPA-induced senescence is dependent on AP-1 activity. **a** Venn diagram identifies genes significantly upregulated upon VPA exposure (Green, Vehicle vs VPA) but differentially repressed by Rapamycin (Purple, VPA vs Rapamycin). **b** Reactome enrichment analysis of the overlapping gene group identified in (**a**). Bars indicate the REACTOME pathways identified, counts show the number of genes. **c** heatmap expression of the top 7 significantly differentially expressed genes of the 233 previously identified. **d** Interactome map of the shortlisted genes identified and relationship to mTOR. Known interactions bars: Blue, from curated database; Pink, experimentally determined; Yellow, text mining; Black, co-expression; Purple, protein homology. **e** Graph showing the percentage of mMaple positive SCOs over time during exposure to the indicated compounds. Organoids were imaged every day and two to 5 fields of view showing 4–12 organoids per field per condition per day were evaluated for the presence of photoconverted (mMaple positive) cells within each organoid. Two-way ANOVA analysis attributed a significant variation to time and treatment (P < 0.0001). Multiple comparison significance of VPA group to other groups per each timepoint is shown on the graph. **f** SA-β-galactosidase assay was performed on organoid sections. Each data point in the whisker box plot represents the percentage of β-galactosidase positive cells in a single section analysed. 7-9 dividual organoids were analysed per condition. **g** Representative fields of sections of organoids after multiple treatments immunolabelled for p21^CIP1/WAF^ and relative quantifications. Scale bar 50 µm. **h** Representative fields of view and quantification of SOX10/GFP positive cells in SCOs after exposure to multiple treatments, white arrows indicate GFP positive cells negative for SOX10; 25-33 individual organoids were analysed per condition, SDs were not significantly different (Brown-Forsythe test), one-way ANOVA with Tukey’s multiple-comparison post hoc corrections, the significance of each comparison is indicated in the graph. Error bars represent s.d. Scale bar 40 µm.

Finally, we aimed to pinpoint the molecular mechanism causing the VPA-induced differentiation of NCC. Starting from our bulk RNAseq analysis (SFig. 8a) we initially identified a substantial effect of Rapamycin on recovering the transcriptional profile induced by VPA exposure (SFig. 8b, c) and found that a number of pathways related to CNS development and senescence were altered (SFig. 8e). We next performed key-driven analysis and the 83 genes overexpressed after VPA exposure but repressed by Rapamycin (*padj* < 0.05, Fig. 6a) which pointed to the involvement of transcriptional regulators (Fig. 6b). Then we identified, among the ten most significantly affected genes, a pool of six targets (EGR1, EGR2, EGR3, FOS, FOSB, JUNB) carrying the potential to cause substantial transcriptional changes [39] and that are restored to basal level by Rapamycin (Fig. 6c). Using STRING [40] we discovered that mTOR and these factors are closely associated on multiple levels through FOS (Fig. 6d and SFig. 8d) and point to a major involvement of the AP1 (Fos/Jun) pioneer factor. Importantly we verified that other proteins belonging to the Fos/Jun network follow a similar transcriptional pattern after exposure to VPA and Rapamycin (SFig. 8f). To confirm that AP1 is necessary for VPA induced senescence, we exposed SCOs to VPA and VPA + SR11302, a non-retinoid specific inhibitor of AP1 (iAP1) that does not alter the activity of VPA as histone acetylase inhibitor (SFig. 8g). We found that specific AP1 inhibition prevented the loss Sox10/mMaple organoids (Fig. 6e), caused a sharp decrease in sen-β-Galactosidase activity (Fig. 6f) and p21^CIP1/WAF^ positive cells (Fig. 6g) and prevented the loss of SOX10+ cells (Fig. 6h) similarly to Rapamycin. However, EVX1+ interneurons were not rescued (SFig. 8h). Altogether these findings suggest that AP1 is the key element that is driving the VPA induced senescence, which can affect specifically the differentiation of neural crest cells.

## Materials and methods

### Plasmids

We inserted a reporter construct into WTC iPCs to express a mMaple-P2A-fLPo cassette [18] in the 3’ untranslated region of *SOX10*. pSpCas9(BB)-2A-Puro (PX459) V2.0 was a gift from Feng Zhang (Addgene plasmid # 62988) was modified to target the region of interest. The fLPo recombinase target used for lineage tracing purposes was a gift from Prof Ryan Lister [20].

### Cell culture and reagents

The WTC iPCs cell line was maintained in mTesr plus (100-1130, Stemcell Tech) after coating with Matrigel (354277, Stemcell Tech). Plasmidic constructs were delivered by nucleofection (P5 Primary Cell 4D, 197187 Lonza). Edited cells were enriched by a temporary incubation with 0.5 ug/ml Puromycin (Gibco). Single clones were generated by seeding single cells in presence of CloneR (05888, Stem Cell Tech). All clones and the bulk population were routinely subjected to mycoplasma testing. Schwann cell precursors (SCPs) were generated by exposing cells to a cocktail of SB-431542 10 μM (1614, Tocris), CHIR99021 3 μM (4423, Tocris), holo-transferrin 10 μg/ml (T0665 Sigma), heregulin 10 ng/ml (78071, Stemcell Tech) Ascorbic acid (50 μg/ml), bFGF 8 ng/ml (233-FB, R&D Systems) in DMEM F12 (11320033, Thermo Fisher), 2% B27 (Gibco, 17504-044), 1% Glutamax (35050061, Thermo Fisher Scientific) 1% MEM-NEAA (Gibco, 11140-050), 0.2% 2-Mercaptoethanol (Gibco, 21985), 1% penicillin and streptomycin (Life Tech), for 3 weeks. SCPs were plated on Matrigel coated multiwell at 10000cell/cm^2^ density and induced to differentiate into MSCs by changing the medium composition to low glucose DMEM (Thermo Fisher, 11885084), 10% FBS (Gibco), 1% penicillin and streptomycin for three weeks. Expression of MSCs markers was confirmed using the FACS Human MSC Analysis Kit (AB_2869404, BD Bioscience) according to the manufacturer instruction. Cells were analysed with a BD LSRFortessa followed by FlowJo analysis. The resulting MSCs were further differentiated to osteoblast and adipocytes using complete osteogenic media: DMEM low glucose (11885084, Gibco), 10% foetal bovine serum (Gibco), 1% penicillin-streptomycin, 100 nM Dexamethasone (265005, Merk) + 100 µM Ascorbic acid 2-phosphate (699004, Sigma) + 10 mM β-glycerolphosphate (G6501, Sigma), and complete adipogenic medium: α-MEM (A1049001, Thermo Fisher Scientific) supplemented with 0.5 mm isobutylmethylxantine (IBMX, I5879, Sigma), 1 μm dexamethasone, 10 μm insulin (91077 C, Sigma) and 200 μm indomethacin (405268, Merk), respectively.

Spinal cord organoids were generated as per [22] with minor modifications. Small iPSC colonies were plated and after 24 h exposed to N2 mesodermal differentiation media 1% N2 (17502001, Gibco), 2% B27, 1% MEM NEAA, 0.1% 2-Mercaptoethanol, 1% penicillin and streptomycin, supplemented with SB-431542 10 μM (1614, Tocris) and CHIR99021 3 μM (4423, Tocris). After 3 days, colonies were detached by dispase incubation and grown in suspension in N2 differentiation media. The medium was supplemented daily with bFGF 20 ng/ml (233-FB, R&D Systems), RA 1 µg/ml (R2625, Sigma), VPA 1 mM (OOAU00071, Sapphire bioscience), Rapamycin 200 nM (US1553210, Merk), iAP1 SR11302 1 µM (2476, Tocris) unless otherwise specified. All experiments were carried out in accordance with the ethical guidelines of the University of Queensland and with the approval by the University of Queensland Human Research Ethics Committee (approval number 2019000159).

### Organoid sectioning and histology

Spinal cord organoids were fixed in 4% paraformaldehyde (PFA) for 1 h at 4 °C and washed with phosphate-buffered saline (PBS) three times for 10 min each at RT before allowing to sink in 30% sucrose at 4 °C overnight. Organoids were next embedded in OCT (Agar Scientific, cat. #AGR1180) and cryosectioned at 14 μm with a Thermo Scientific NX70 Cryostat. Tissue sections were used for immunofluorescence and for the SA-β-Gal assay. For immunofluorescence, sections were blocked and permeabilized in 0.1% Triton X-100 and 3% Bovine Serum Albumin (BSA) in PBS followed by incubation in a humid chamber with primary antibodies overnight at 4 °C, washed three times with PBS 0.1% Triton X-100 and incubated with secondary antibodies for 1 h at RT. 10 ng/ml DAPI (Sigma, cat. #D9564) was added for 5 min to mark nuclei. Secondary antibodies conjugated to Alexafluor 488, 568, or 647 (Invitrogen) were used for detection. For wholemount staining organoids were permeabilized by overnight incubation in PBS 0.5% Triton X-100 at 4 °C, followed by the same immunostaining protocol.

### Zebrafish wholemount

Zebrafish were maintained in standard housing conditions as described in [41]. Tupfel long-fin wild-type embryos were collected at 0 h post fertilization (hpf) and exposed to standard embryo medium (control) 150 µM VPA or 150 µM VPA and 200 nM Rapamycin for 48 h. Embryos were then rinsed in embryo medium, manually dechorionated, and fixed in 4% PFA at 4 °C overnight. Samples were incubated with primary antibody (anti-Sox10, 1:200) overnight at 4 °C, washed, and incubated with secondary antibody (Alexa 488, 1:500) for 4 h at RT. Immediately prior to imaging, embryos were mounted on slides in 2% low melting temperature agarose.

### Imaging and analysis

Immunofluorescence images were acquired using a Zeiss LSM 900 Fast Airyscan 2 super-resolution microscope, Zeiss AxioScan Z1 Fluorescent Imager, PerkinElmer Operetta CLS High Content Analysis System, and Yokogawa W1 Spinning Disk Confocal for zebrafish embryos. Images were analysed with a custom pipeline compiled on CellProfiler or ImageJ. Cells in each frame of the time-lapse were segmented using the AI-based software CellPose [25] using the cytosolic GFP channel signal. The position (centroid) of the segmented cells in each frame were used for cell tracking by applying the Hungarian linker algorithm through custom-made MATLAB software, as described in [26].

### Molecular analysis

For western blot analysis samples were lysed in RIPA buffer (Thermo Fisher) followed by one sonication pulse (1” at 40% intensity, VCX750, Sonics). The protein content was assessed by BCA assay (23225, Thermo Fisher). 50 to 100 ug protein was loaded in each lane and separated on 4–20% precast polyacrylamide gel (17000436, Biorad) and transferred on PVDF membrane (1704156, Biorad) using Transblot Turbo (Biorad) programme 1. The membrane was blocked with 5% skim milk in TBS-T for 1 h and incubated with primary antibodies overnight at 4 °C, washed and incubated with secondary antibodies for 1 h at RT. After brief exposure to ECL substrate (1705060, Biorad) the luminescent signal was detected with a ChemiDoc MP Imaging system (Biorad).

### Antibodies

SOX10 (89356, NEB, 1:400), sox10 zebrafish (ab229331, Abcam, 1:200), BRN2, p21^CIP1/WAF^ (2946, NEB, 1:3000), Isl1/2 (67.4E12, DSHB, 1:50), EVX1(ab220665, Abcam, 1:500), OP (ab8448, Abcam 1:1000), FABP4 (ab92501, Abcam 1:500), B-Actin (A3864, Sigma, 1:10000), ERK (9102, NEB, 1:10000); phospo-ERK (9101, NEB, 1:10000); AKT (9272, NEB, 1:5000); Phospo-AKT (9271, NEB, 1:5000); cFOS (134122, Abcam, 1:2000), ; anti-rabbit IgG (Invitrogen, A10042, 1:500); anti-rabbit IgG (Invitrogen, A21245, 1:500); anti-mouse IgG (Invitrogen, A11029, 1:500); anti-mouse IgG (Invitrogen, A21235, 1:500).

### RNA isolation

RNA from cells or spinal cord organoids was extracted with Nucleospin RNA (Scientificx) according to the manufacturer’s instructions. RNA integrity was evaluated with the 2100 Bioanalyzer RNA 6000 Pico Chip kit (Agilent) using the RNA Integrity Number (RIN). RNA samples with a RIN > 7 were considered of high enough quality for bulk RNA sequencing.

### Bulk RNA sequencing

Messenger RNA was purified from total RNA using poly-T oligo-attached magnetic beads. After fragmentation, the first strand cDNA was synthesized using random hexamer primers. Differential expression analysis between conditions was performed using the DESeq2 R package (1.20.0). The resulting P-values were adjusted using the Benjamini and Hochberg’s approach for controlling the false discovery rate. Genes with a *Padj* <=0.05 found by DESeq2 were assigned as differentially expressed. Gene Ontology (GO) enrichment analysis of differentially expressed genes was implemented by the cluster Profiler R package, using online tools [42], or plotted by https://www.bioinformatics.com.cn/en.

### Real-time quantitative PCR

In total, 1–0.5 μg of total RNA was reverse transcribed using iScript cDNA Synthesis Kit (Bio-Rad). A volume corresponding to 5 ng of initial RNA was employed for each real-time PCR reaction using PowerUp SYBR Green Master Mix (Applied Biosystems) on a CFX Opus Real-Time PCR detection system. Glyceraldehyde-3-phosphate dehydrogenase (GAPDH) was used as normaliser. Primers sequences (5’-3’ orientation) are listed in STable 1.

### Statistical analysis

The experimental design illustrated in Fig. 3a was employed to determine the appropriate sample size for subsequent analyses. Utilizing this design, the sample size was calculated to be *n* = 3, ensuring that the resulting confidence interval would maintain a margin of error within 0.05 at the 95% confidence level. Results are shown as mean ± standard deviation (s.d.) or Box Plot as median and whisker. P value was calculated by the indicated statistical tests on Prism software. In the figure legends, n indicates the number of independent experiments or biological replicates when not specified in results.

## Discussion

In this study we show for the first time that senescence is a mediator of VPA-associated teratogenicity in a developing human spinal cord model. Importantly we demonstrate in vitro and in vivo that VPA exposure severely affects the normal differentiation trajectory of neural crest cells, a cell population of pivotal importance during neurodevelopment, and this process can be specifically prevented by suppressing senescence with the senostatic Rapamycin.

Epilepsy is diagnosed in >5 million people each year, and VPA is one of the most widely adopted and most effective anti-seizure medications, in particular for people with generalised epilepsies [2, 3]. Despite the teratogenic effects of VPA being a major limitation to its use in clinical practice in women of childbearing potential with epilepsy, very little is known of the underlying mechanisms. Features of VPA teratogenicity include neural tube defects, neurocognitive and neurobehavioural deficits, congenital heart defects, peculiar facial features, urogenital malformations and musculoskeletal abnormalities [43]. Thus, we decided to focus our attention on Neural Crest Cells (NCCs), which arise during the first stages of neural tube development and contribute to the development of most tissues affected in FVS.

In order to dissect the mechanisms for the teratogenic effects of VPA in NCCs, we designed a genetic switch for gene expression reporting/lineage tracing which is turned on by endogenous *SOX10* expression, does not display detectable leakage, and is maintained after multiple passages and across multiple lineages, overcoming limitations to lineage tracing in human cultured iPSC associated with oscillations in the expression of driver genes and inaccurate tracing [44]. This system enabled for the first-time real time single cell tracking of the behaviour of spontaneously formed NCC in a 3D model of human developing neural tube [45]. Our approach has multiple advantages: it simplifies the detection of newly specified NCCs in 3D, identifies differentiation events indicated by the loss of the SOX10 reporter signal, and allows lineage tracing of the differentiated progeny. Using this approach, we confirmed in a human 3D system that RA exposure induces the differentiation of neural crest cells into ISL1+ peripheral neurons. Indeed, animal studies in chick and mouse suggest that NCCs differentiation is driven by a gradient of RA in vivo [23, 46, 47], and our results indicate that our model can successfully recapitulate this process, and thus is suitable for investigating VPA teratogenicity and NCC behaviour.

We observed that exposure to VPA significantly promoted NCC differentiation, switching from a proliferative to a MSC differentiation programme, thus overcoming the proliferative stimuli provided by bFGF. This was accompanied by a significant induction of cellular senescence as shown by a significant increase of multiple senescence markers including p16^INK4/CDKN2^, MYC and TGFβ at transcriptional level, SA-β-Galactosidase activity, and p21^CIP1/WAF^ protein. Altogether our data are in strong agreement with the evolving body of literature supporting the pivotal role of cellular senescence in the precise regulation of developmental processes [48] and aligns with prior research on VPA-induced abnormalities in folding neural tube [32] and brain development [49]. VPA is a histone deacetylase (HDAC) inhibitor, however the role of HDACs in senescence is still controversial, as they are involved in both promoting [50] or hindering senescence [51]. We believe this is most likely explained by the specificity of single HDACs to different epigenetic targets. Interestingly, our results suggest that VPA-induced senescence can be countered despite the HDAC-inhibition activity. Putting together the pieces of the epigenetic puzzle behind senescence is a next fundamental step to understanding the phenomenon of aging.

Since HDAC inhibition has been previously associated with increased SASP expression [52], and to establish a causal relationship between the induction of senescence and altered NCCs differentiation, we aimed to prevent VPA-induced senescence by suppressing SASP expression [53]. Our data suggested induction of cellular senescence through activation of the Akt-mTOR-p53-p21 signalling pathway [54]. Thus we selected Rapamycin, an inhibitor of the mTOR signalling cascade and a senomorphic drug that has shown consistent effects on cellular senescence and substantial efficacy in different aging models [17]. Furthermore, Rapamycin has been shown to prevent premature senescence within the neural tube in diabetic rodent model [55]. Rapamycin strikingly reduced VPA-induced abundance of p21^CIP1/WAF^, a primary mediator of developmental senescence [56], and prevented aberrant VPA-induced differentiation of NCC into MSC. Next, we decided to confirm our findings in an in vivo model of developing animal.

Exposure of zebrafish larvae to VPA is not generally associated with neural tube defects, but rather causes other neurodevelopmental defects such as deformation of eyes and craniofacial abnormalities [57]. Intriguingly, NCCs contribute to the development of these tissues, thus previous observations support our view of a pivotal role of NCC in mediating the teratogenicity of VPA. Our results show that VPA treatment at a concentration within the therapeutic range (50–100 mg/L) [58] severely alters anatomical facial features with a concomitant and significant decrease in Sox10 positive cells. Similar defects are also observed after ablation of HDAC4 [59]. Strikingly Rapamycin could largely prevent these defects, fully supporting our in vitro observations.

Finally, we investigated the molecular cascade leading to NCCs differentiation after VPA exposure. Transcriptome comparisons of vehicle, VPA and Rapamycin treated organoids suggested an involvement of an array of master regulators involved in senescence. Specifically, EGR1, a gatekeeper of inflammation [60], EGR2, regulator of senescence [61] and AP1, a pioneer factor that coordinates the cellular senescence programme at a transcriptional level [62, 63]. Our results indicate that AP1 inhibition can substantially prevent the increase in cellular senescence and supports the preservation of NCC identity but not the differentiation of interneurons. Thus, AP1 is a key mediator of VPA induced senescence and neurodevelopmental teratogenicity, but other processes may still be impacted. Interestingly, retinoids are repressors of AP1 activity [64] and given the presence of RA in an anteroposterior gradient along the developing spinal cord, our results suggest that dose-dependent VPA-induced defects affect most commonly the caudal region (i.e. spina bifida) as consequence of the lower concentration of RA in the area – which is supported by clinical studies [28, 65].

To our knowledge this is the first report linking VPA exposure to a specific alteration in the neural crest differentiation programme. One of the main characteristics of Foetal Valproate syndrome is a distinctive facial appearance, which suggests a disruption of the normal differentiation and migration programme of NCCs contributing to the craniofacial bone. However past literature has mainly focused on the effects of VPA on brain development [66]. Our results revealed a dysregulated senescence programme behind the teratogenic effects of VPA. We reflect that VPA induces multiple major senescence regulators like p16^INK4/CDKN2^, p21^CIP1/WAF^, and EGR2, and postulate that the changes in the levels of senescence and inflammation (SASP and EGR1) suggest an activity of Rapamycin on multiple levels. Nevertheless, we identify the master transcription factor AP1 as a necessary mediator of the observed defects.

After several decades since its initial adoption, Valproic acid continues to demonstrate remarkable efficacy as an antiepileptic drug. Moreover, its application has extended beyond epilepsy management, encompassing other therapeutic contexts such as bipolar disorder. Thus, severe restrictions to its prescriptions will create a set of challenges for health care providers and patients. Collectively, we propose an entirely novel model of VPA’s mechanism of teratogenicity and suggest that co-treatment with Rapamycin could allow safe prescription of VPA to women of childbearing potential in whom it is the most effective medication, in particular those with generalised epilepsies.

## Supplementary information

SFig1

SFig2

SFig3

SFig4

SFig5

SFig6

SFig7

SFig8

STable 1

STable 2

Supplementary Figures legends

Video1

Video2

## Data Availability

All RNA-seq data have been deposited in NCBI’s GEO and are accessible through GEO series accession number GSE241903. All data needed to evaluate the conclusions in the paper are present in the paper and/or the Supplementary Materials.
