# Supplementary figures and images for: Valproic acid-induced teratogenicity is driven by senescence and prevented by Rapamycin in human spinal cord and animal models
Source: Mol Psychiatry. 2024 Sep 3;30(3):986–98. doi: 10.1038/s41380-024-02732-0 (PMC11835743; doi:10.1038/s41380-024-02732-0)

**a**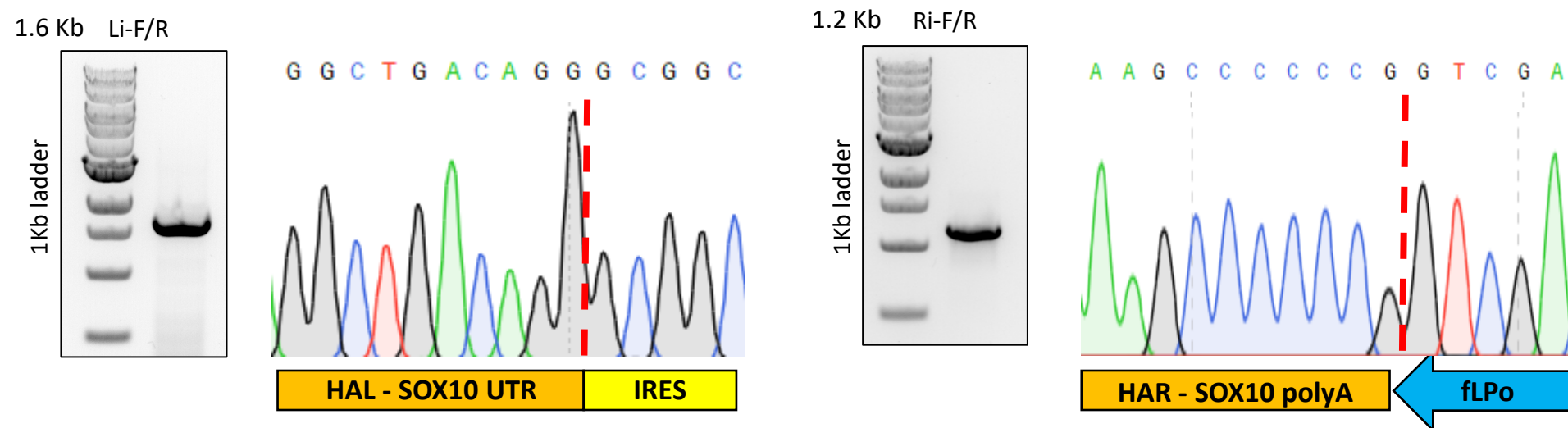**b**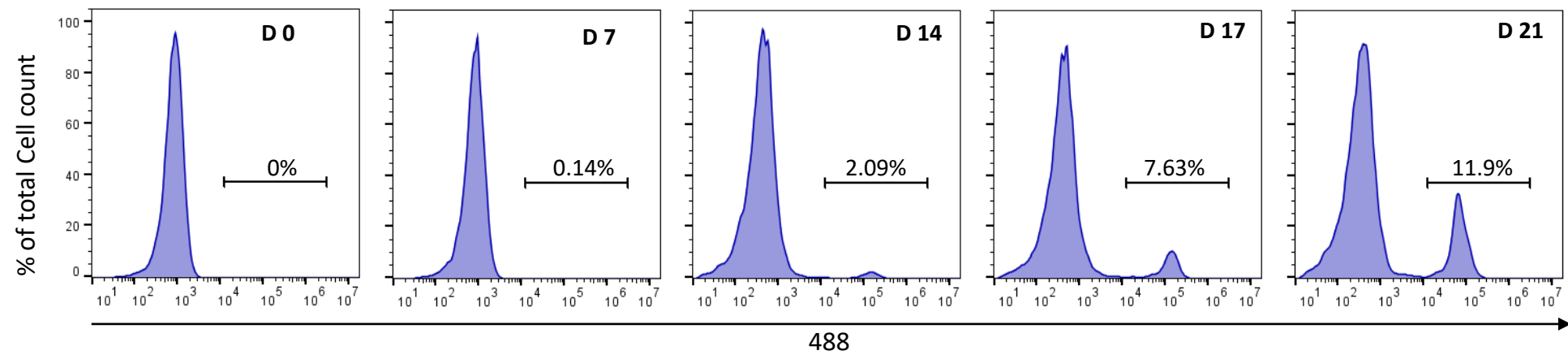**c**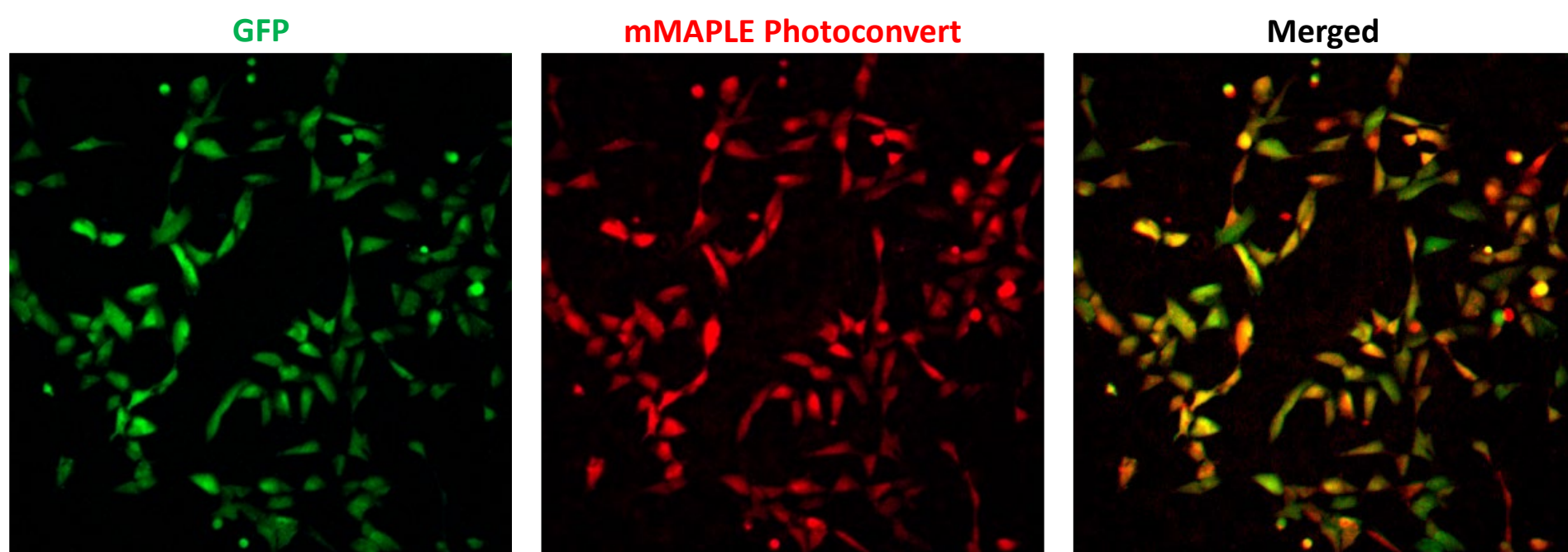**d**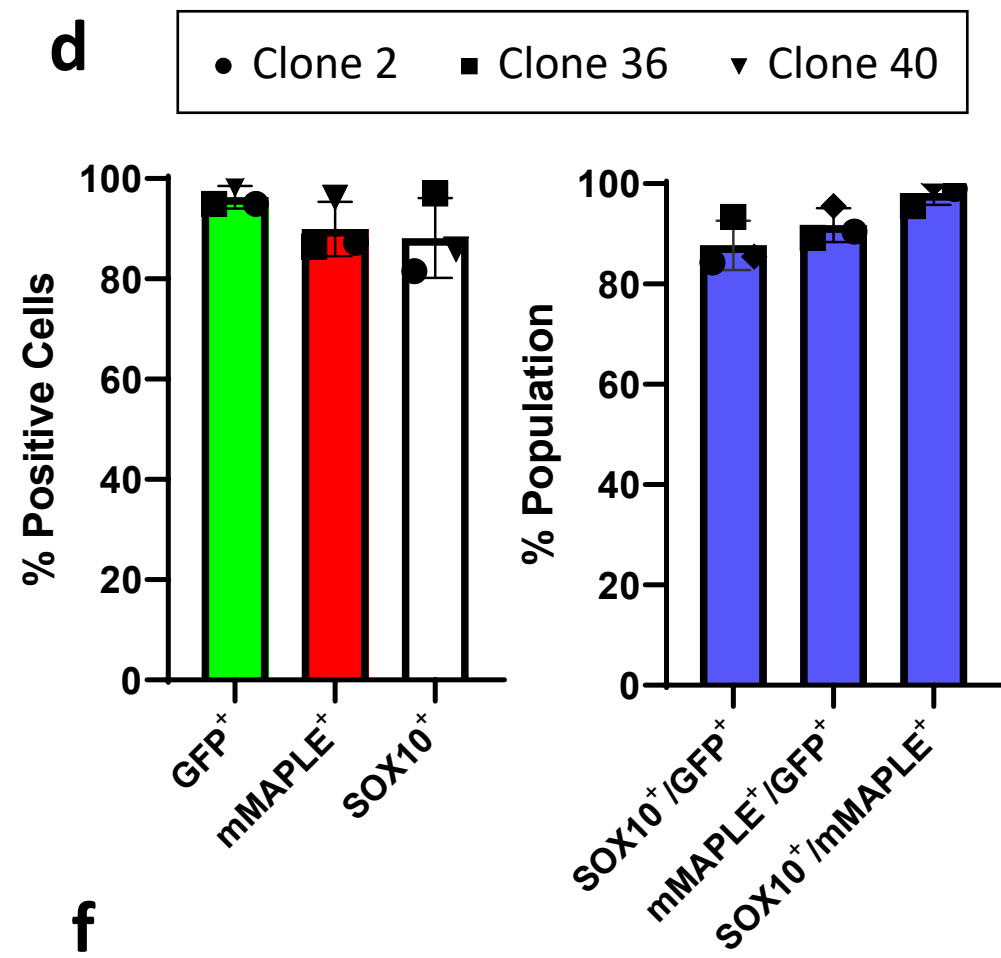**e**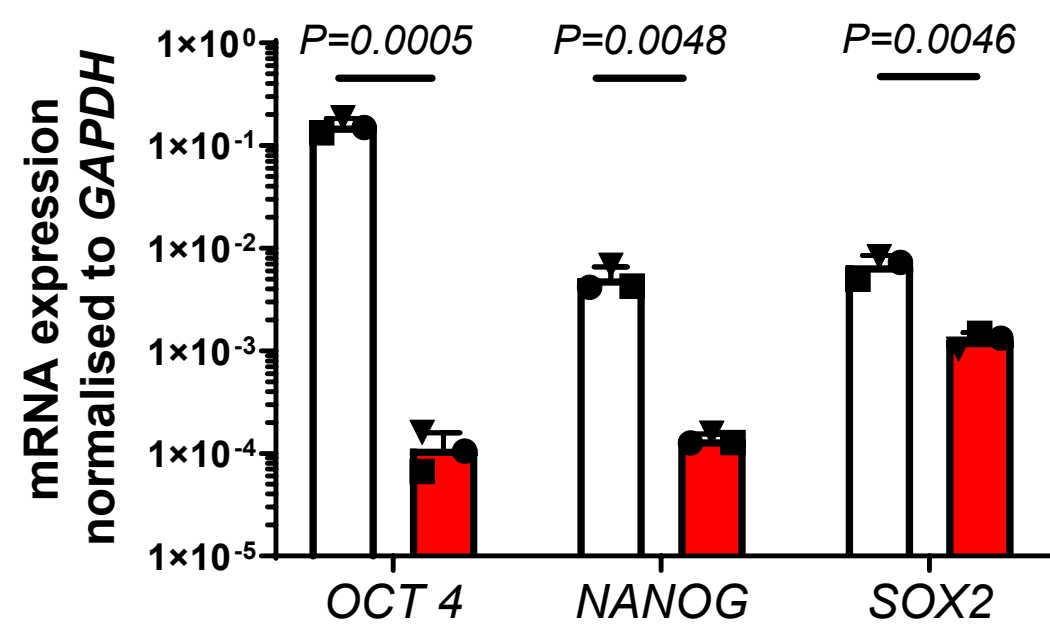**f**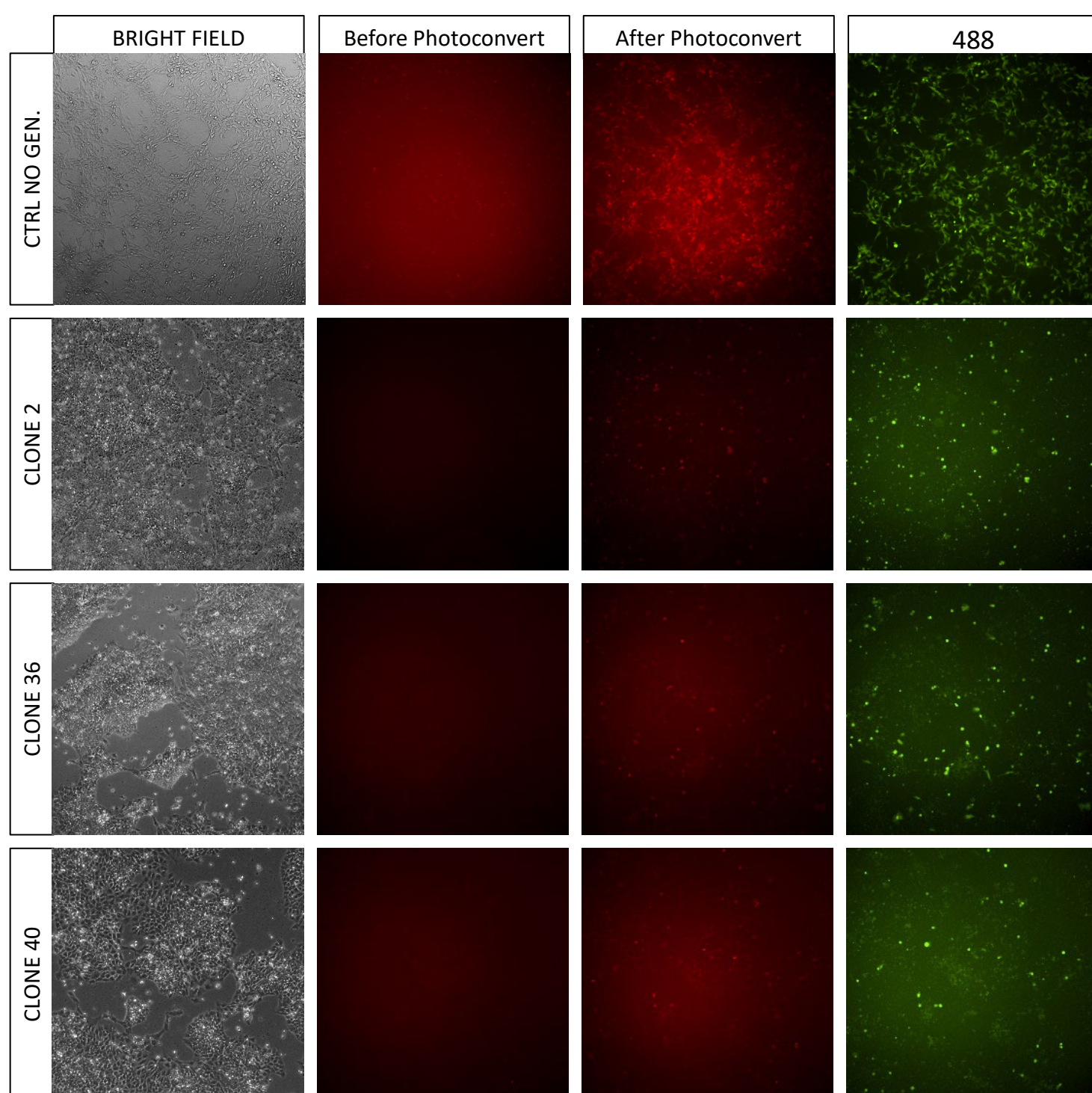

Supplement: Supplementary file 1 — SFig1 [file 41380_2024_2732_MOESM1_ESM.pdf]

a

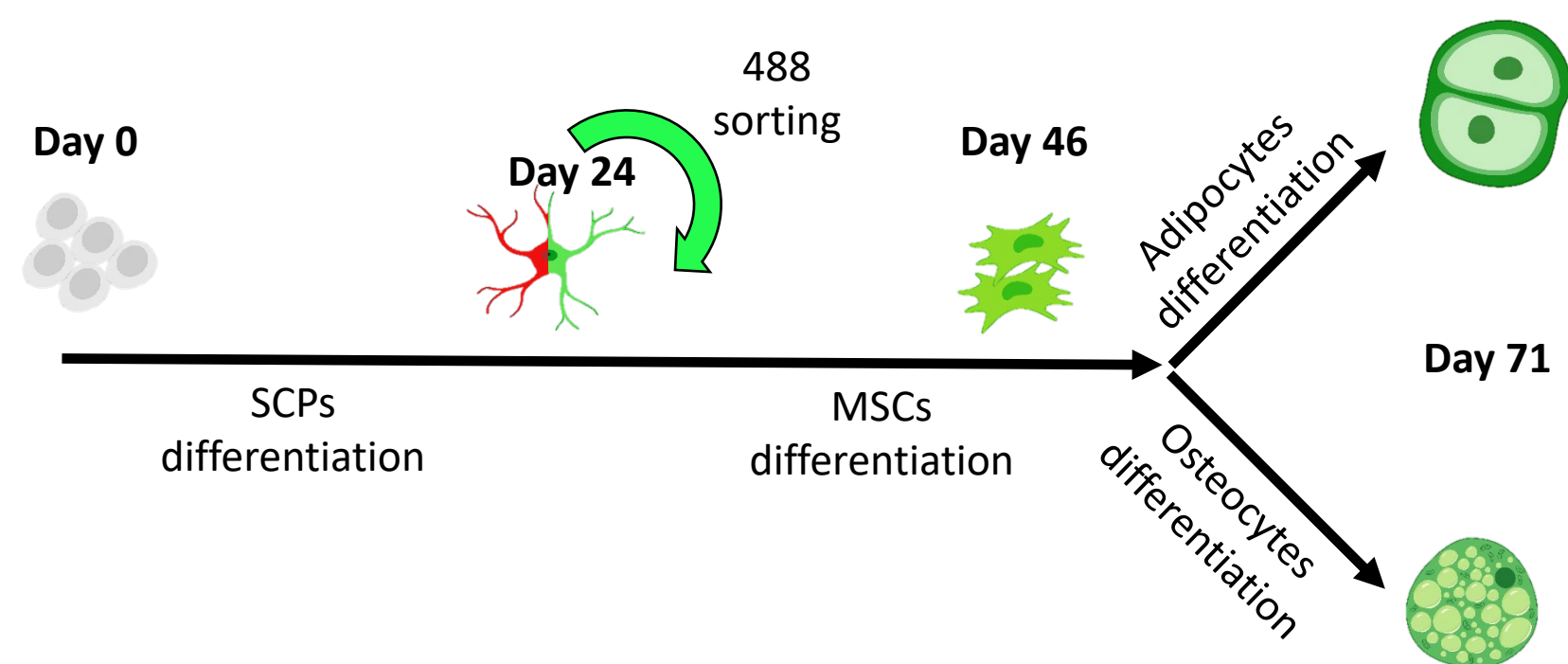

b

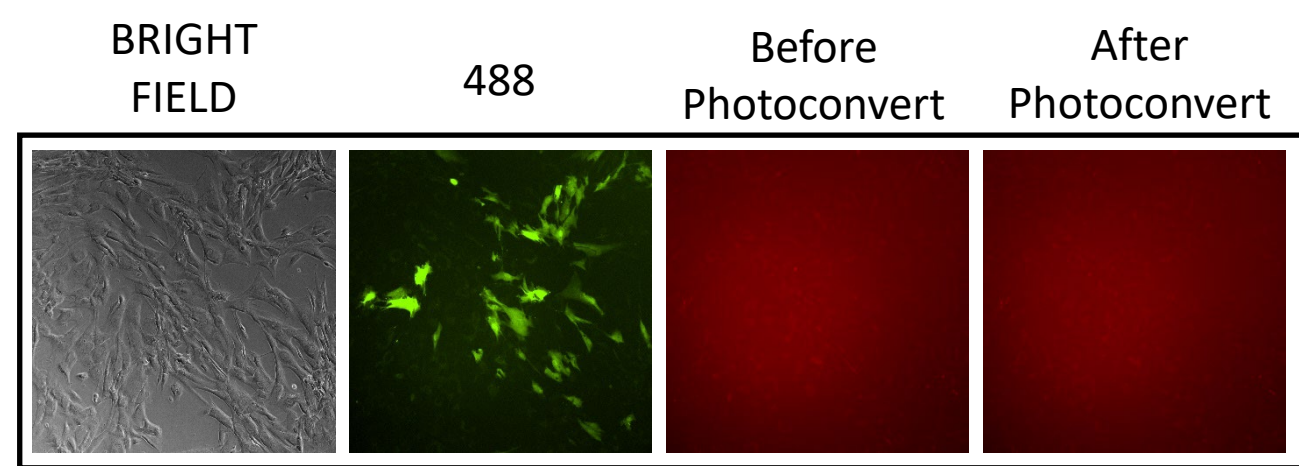

c

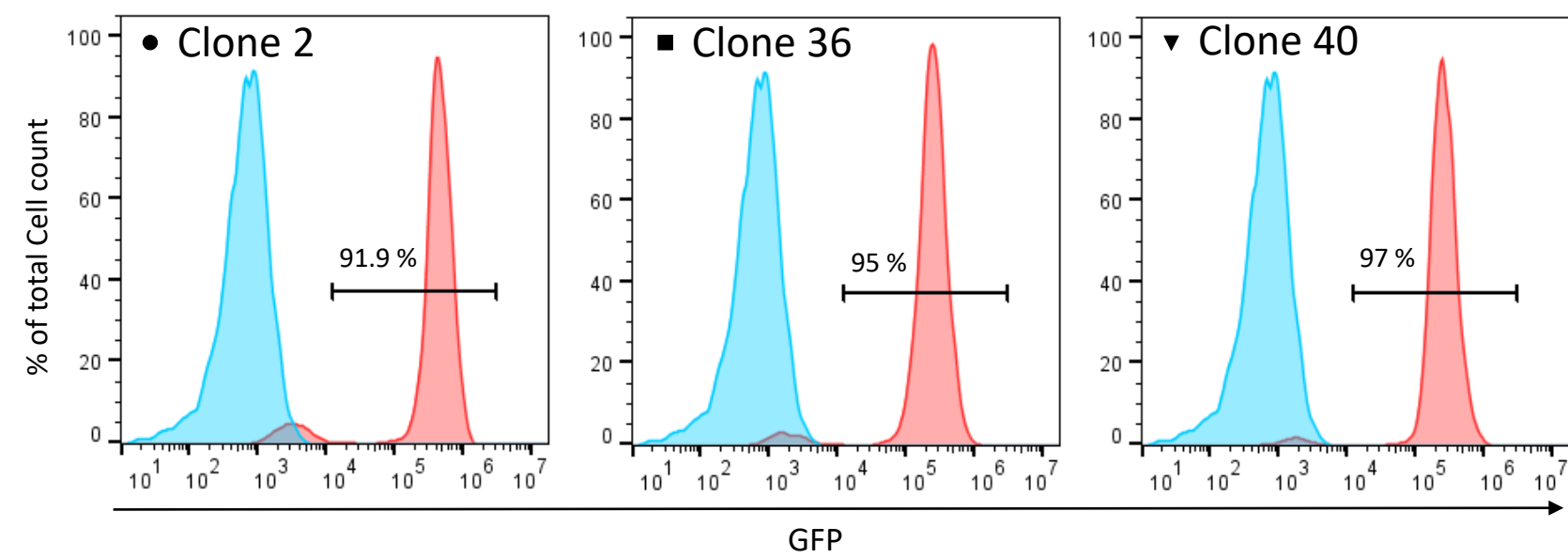

d

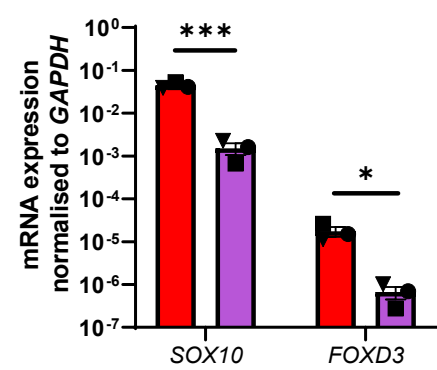

e

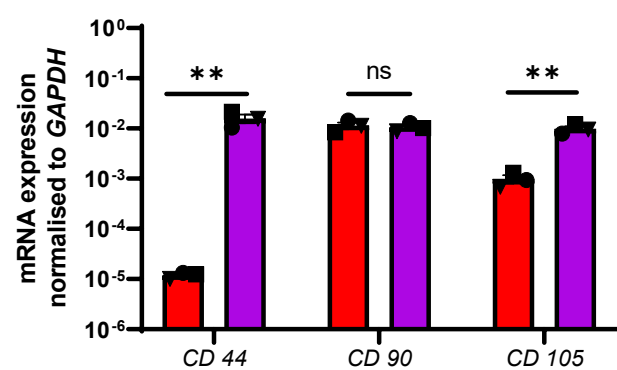

f

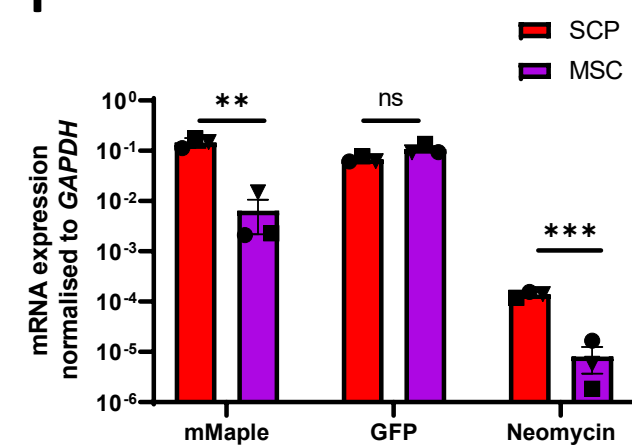

g

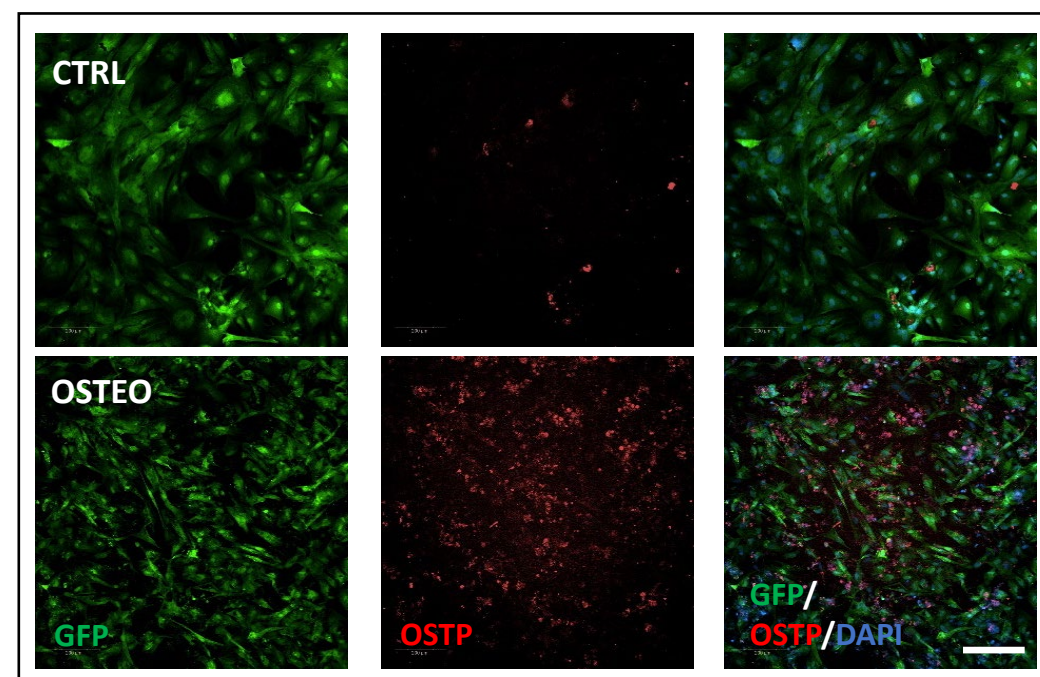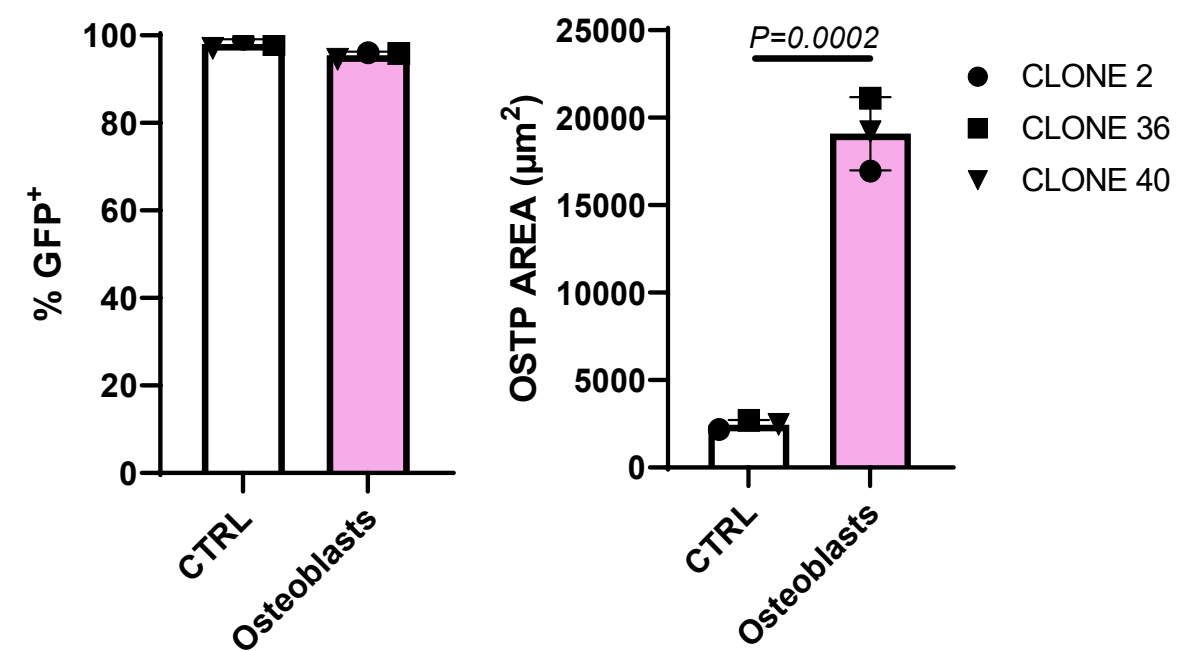

h

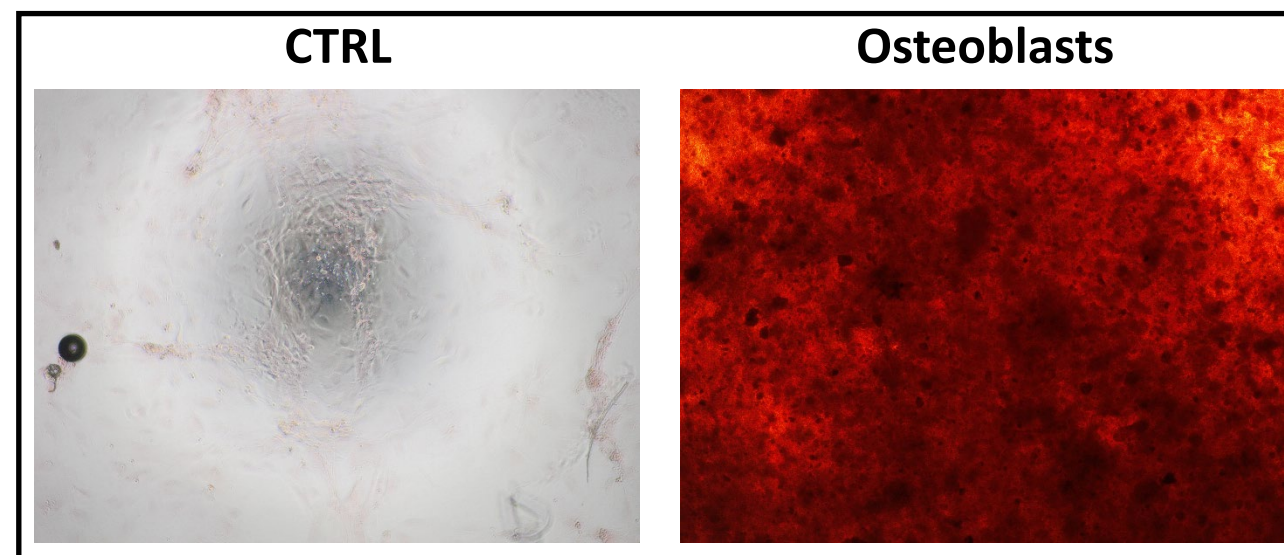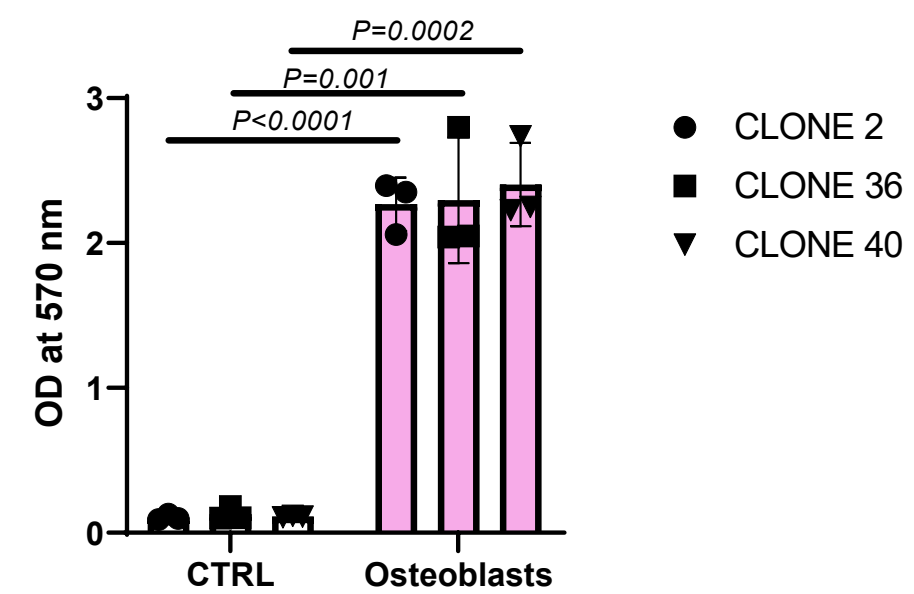

i

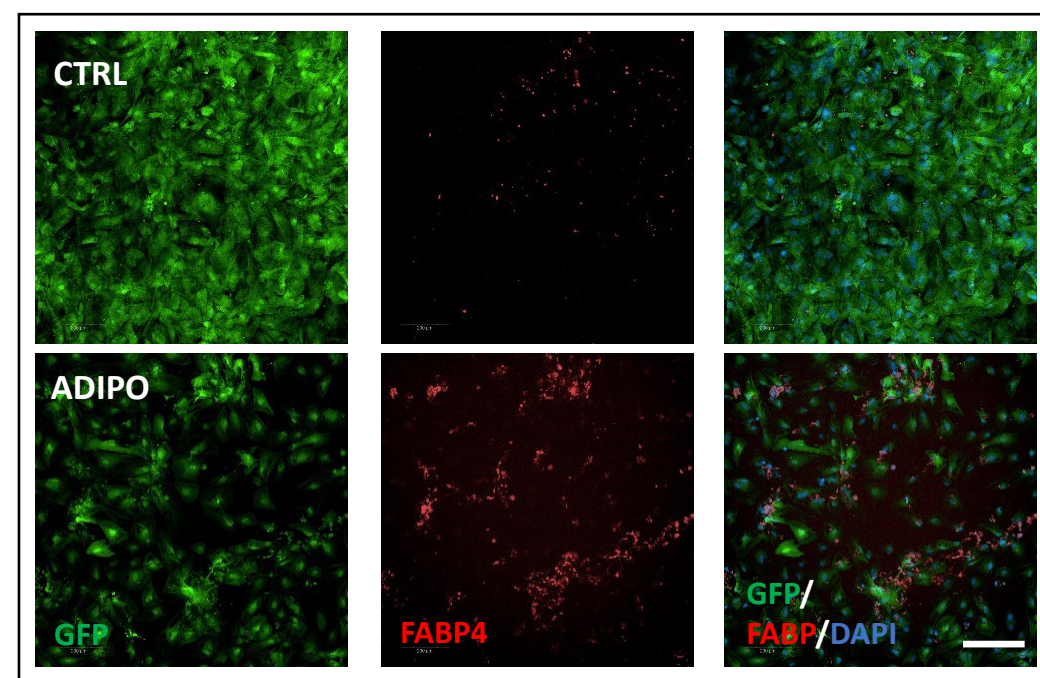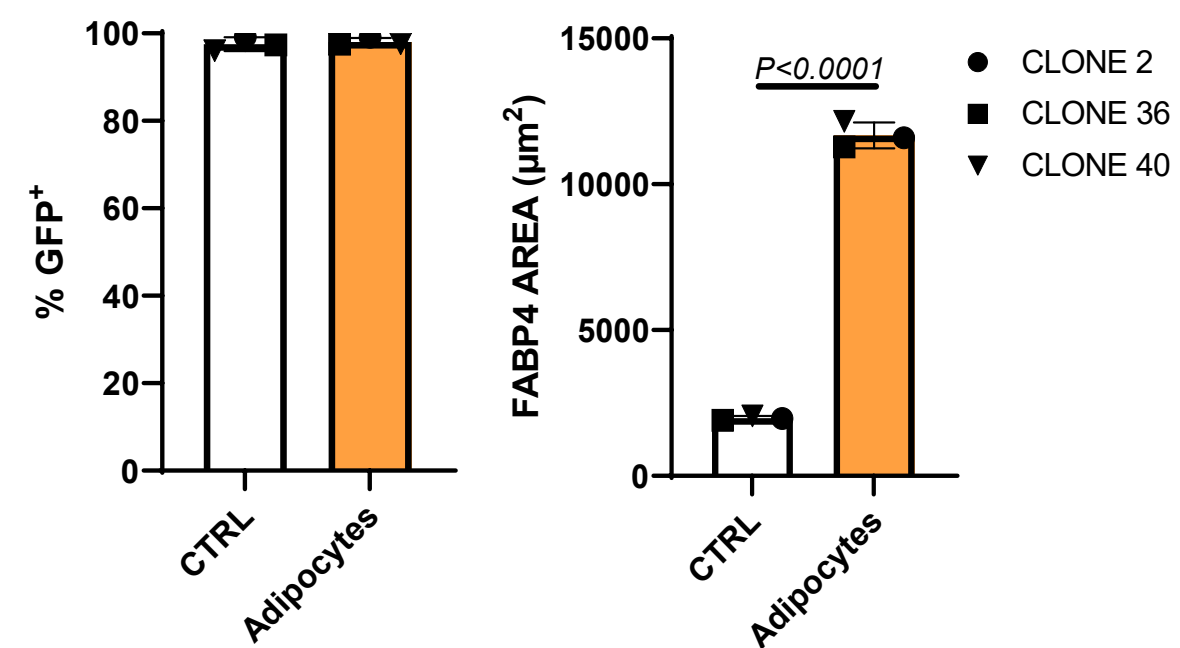

l

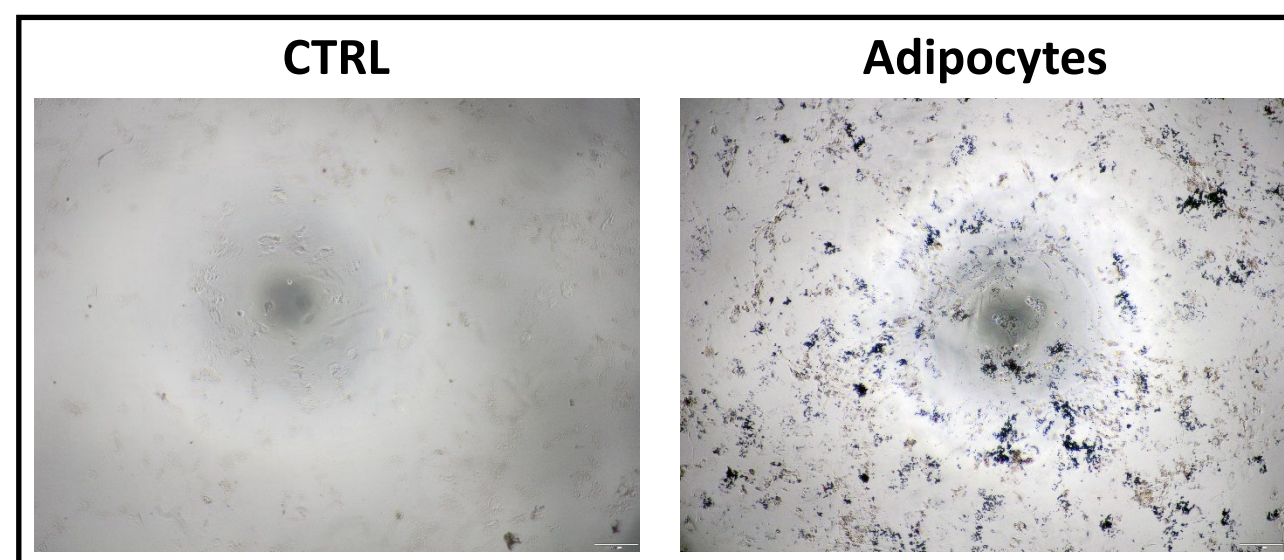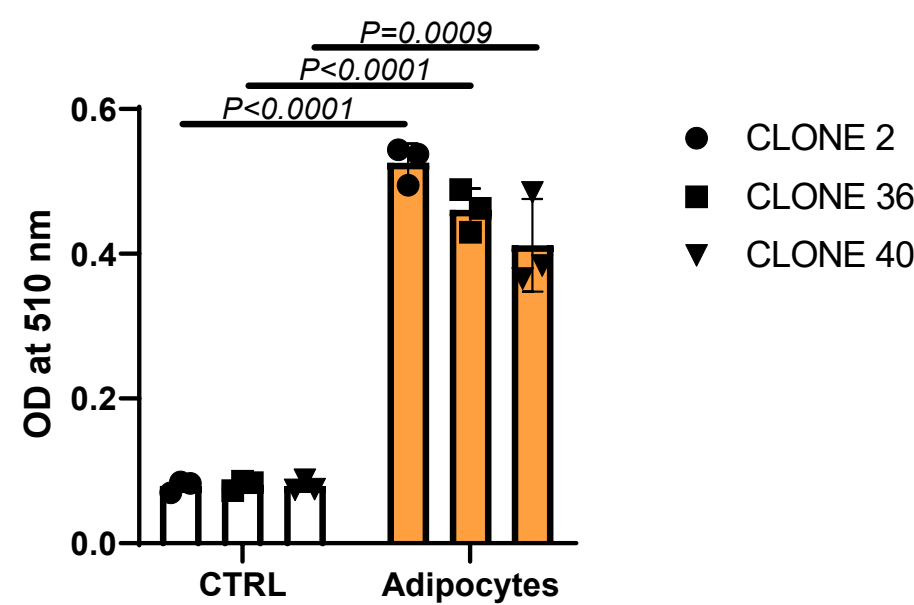

Supplement: Supplementary file 2 — SFig2 [file 41380_2024_2732_MOESM2_ESM.pdf]

**a**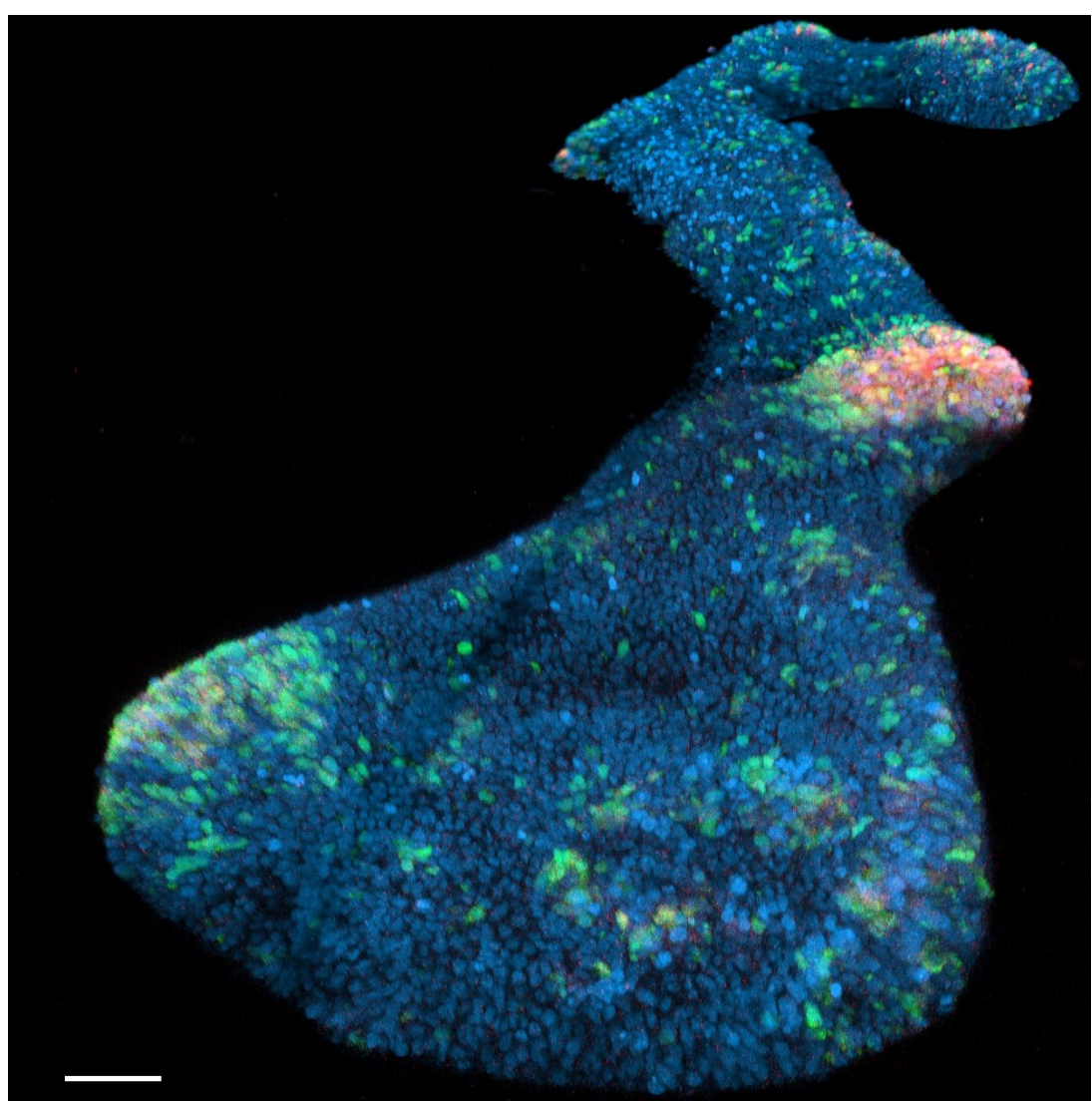**b**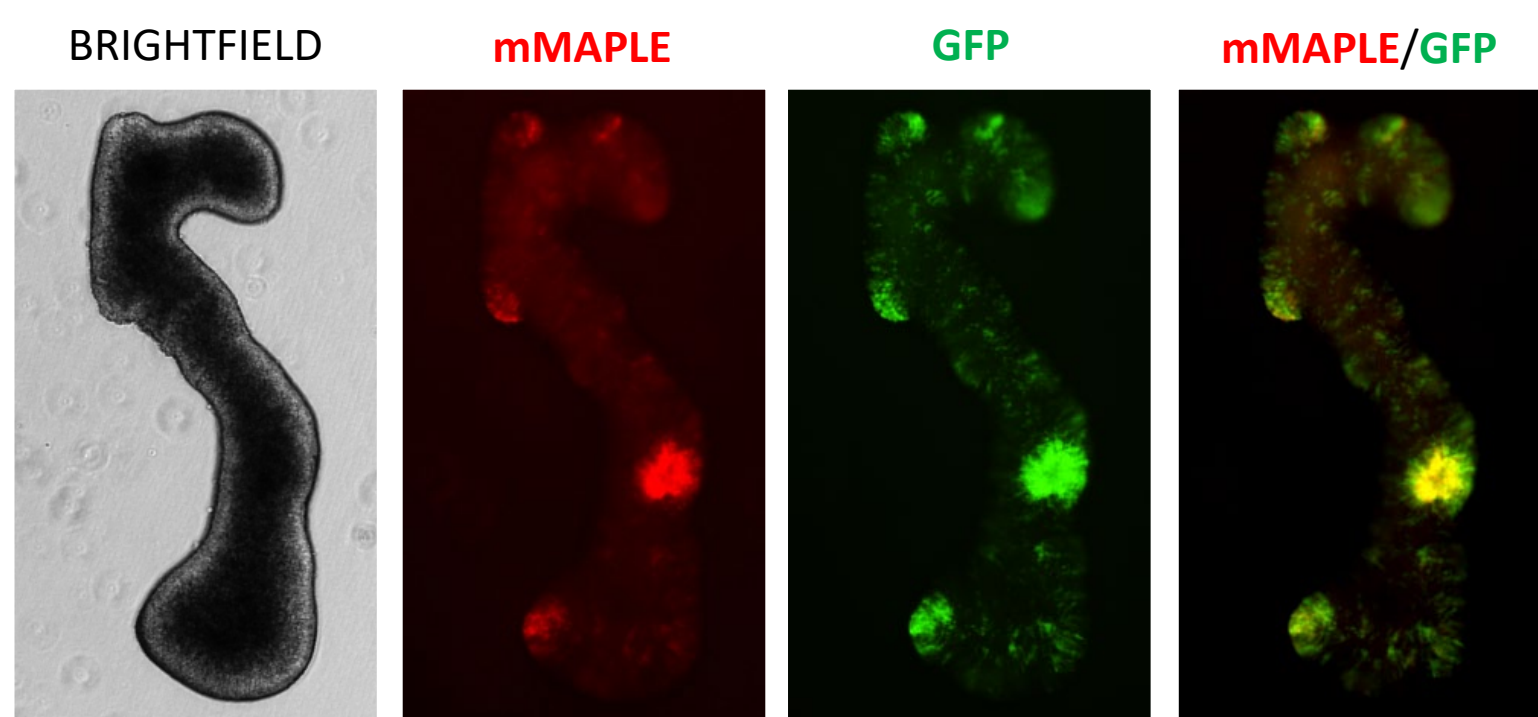**d**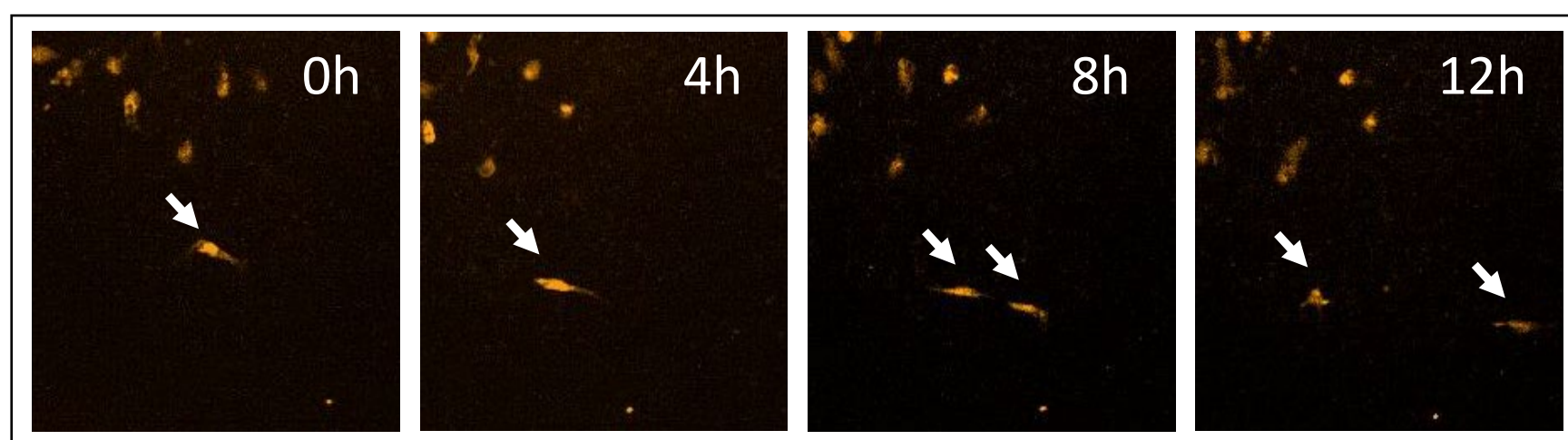**c**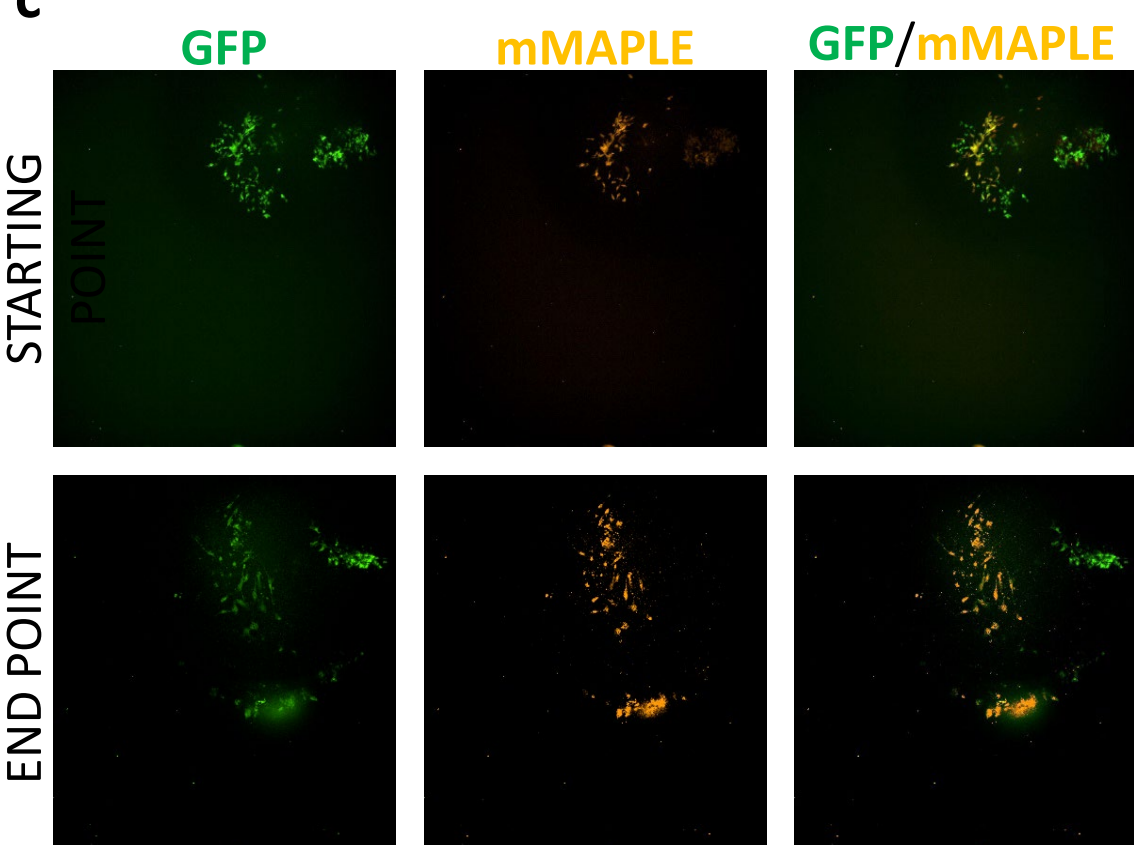**e**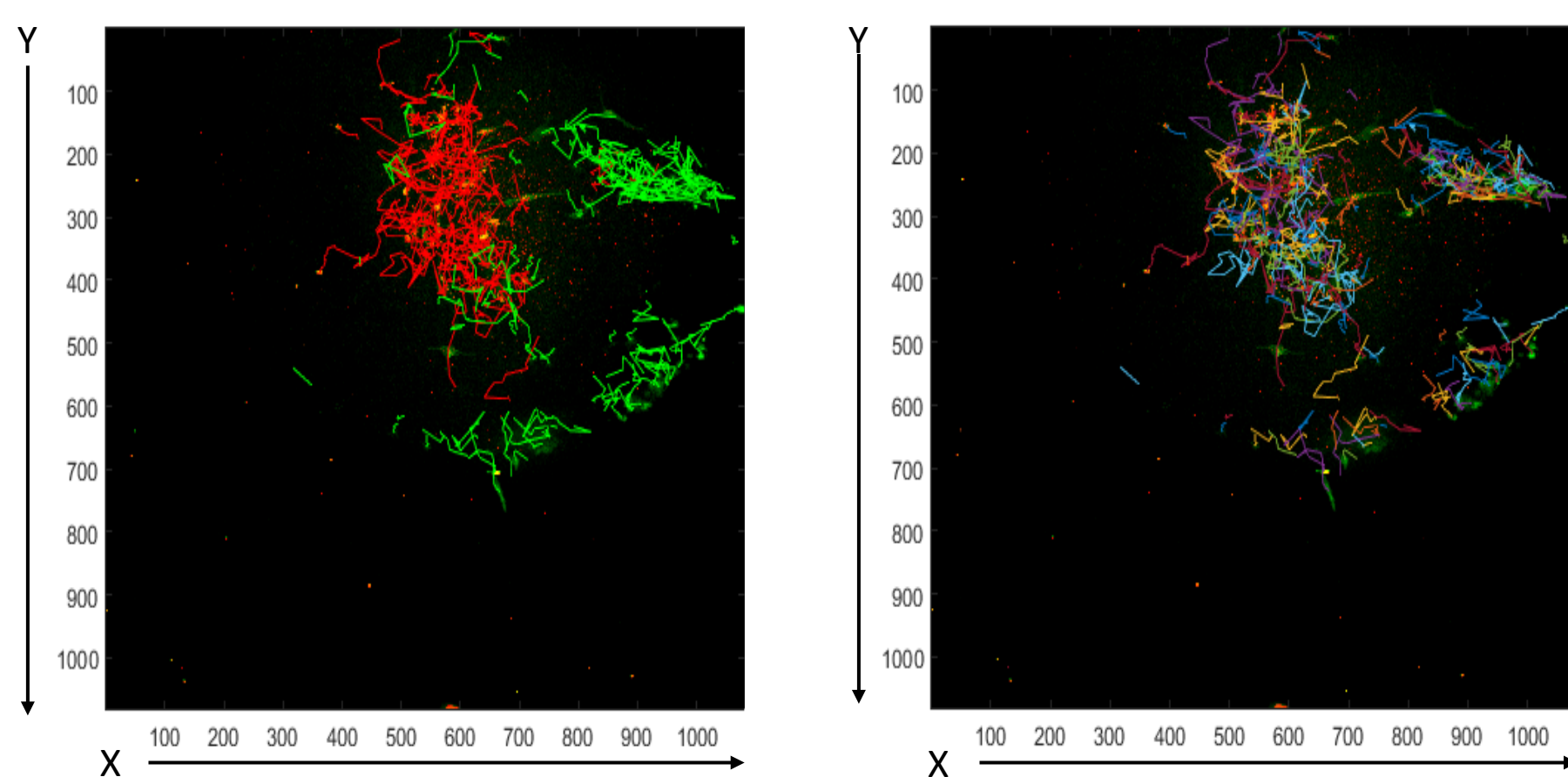**f**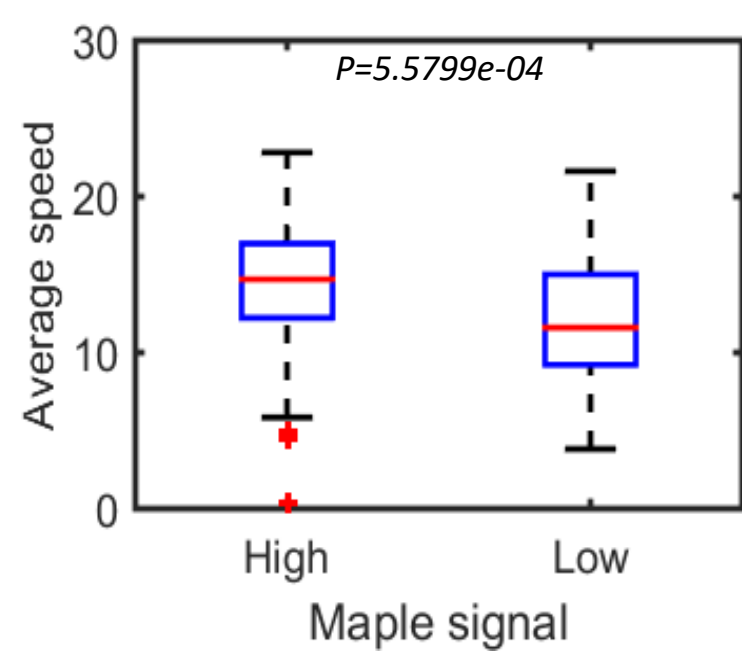**g**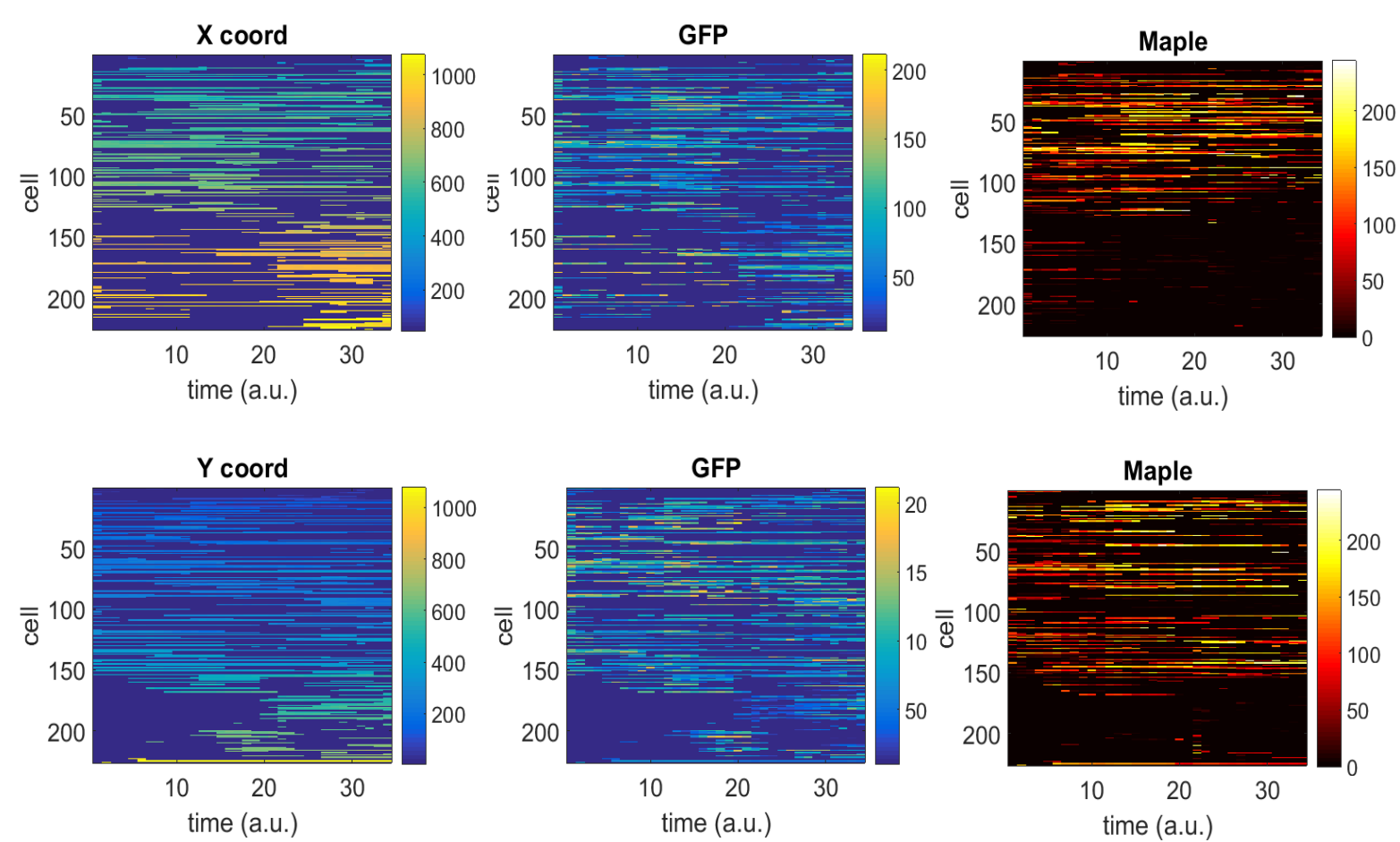

Supplement: Supplementary file 3 — SFig3 [file 41380_2024_2732_MOESM3_ESM.pdf]

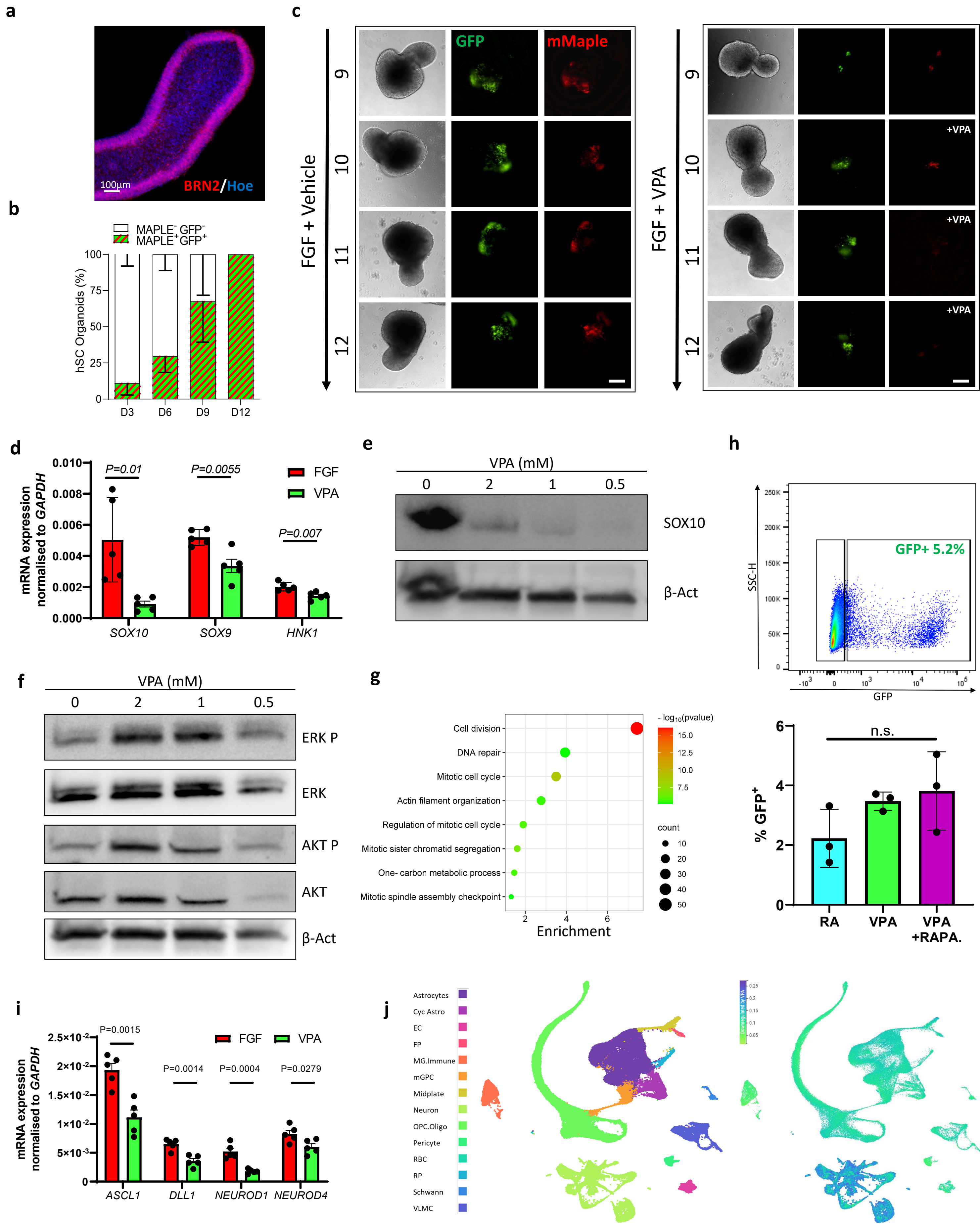

Supplement: Supplementary file 4 — SFig4 [file 41380_2024_2732_MOESM4_ESM.pdf]

**a**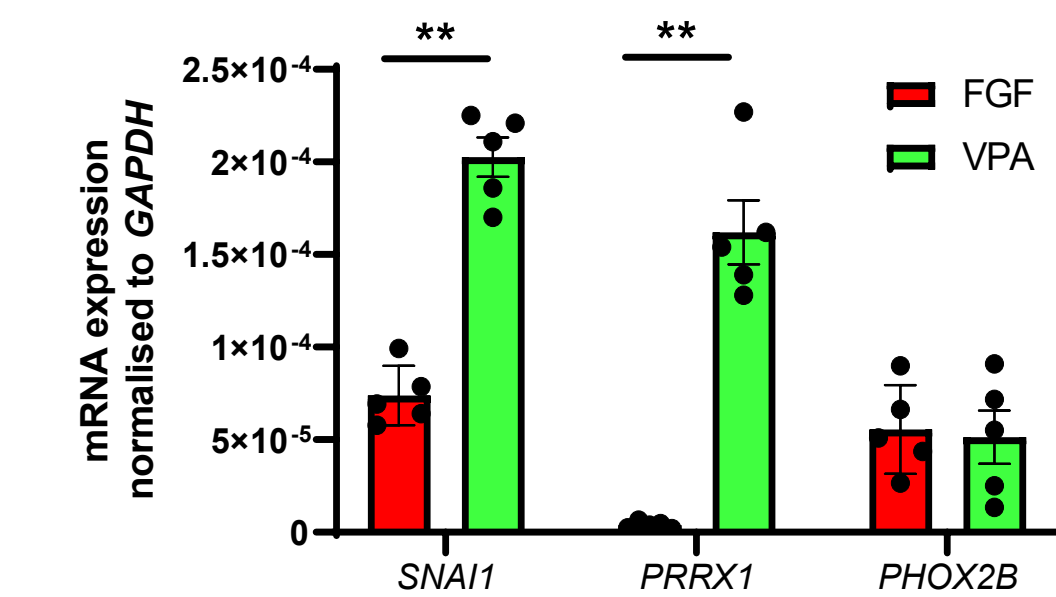**b**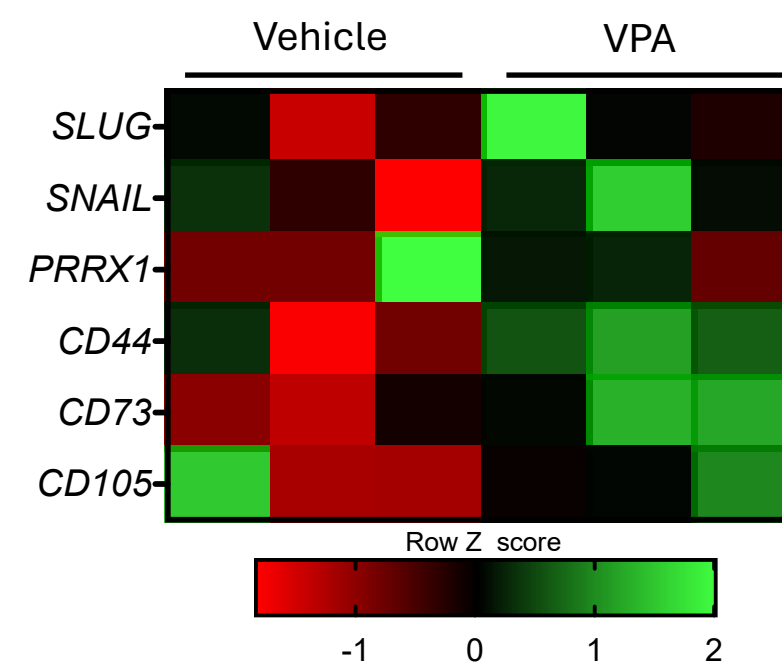**c**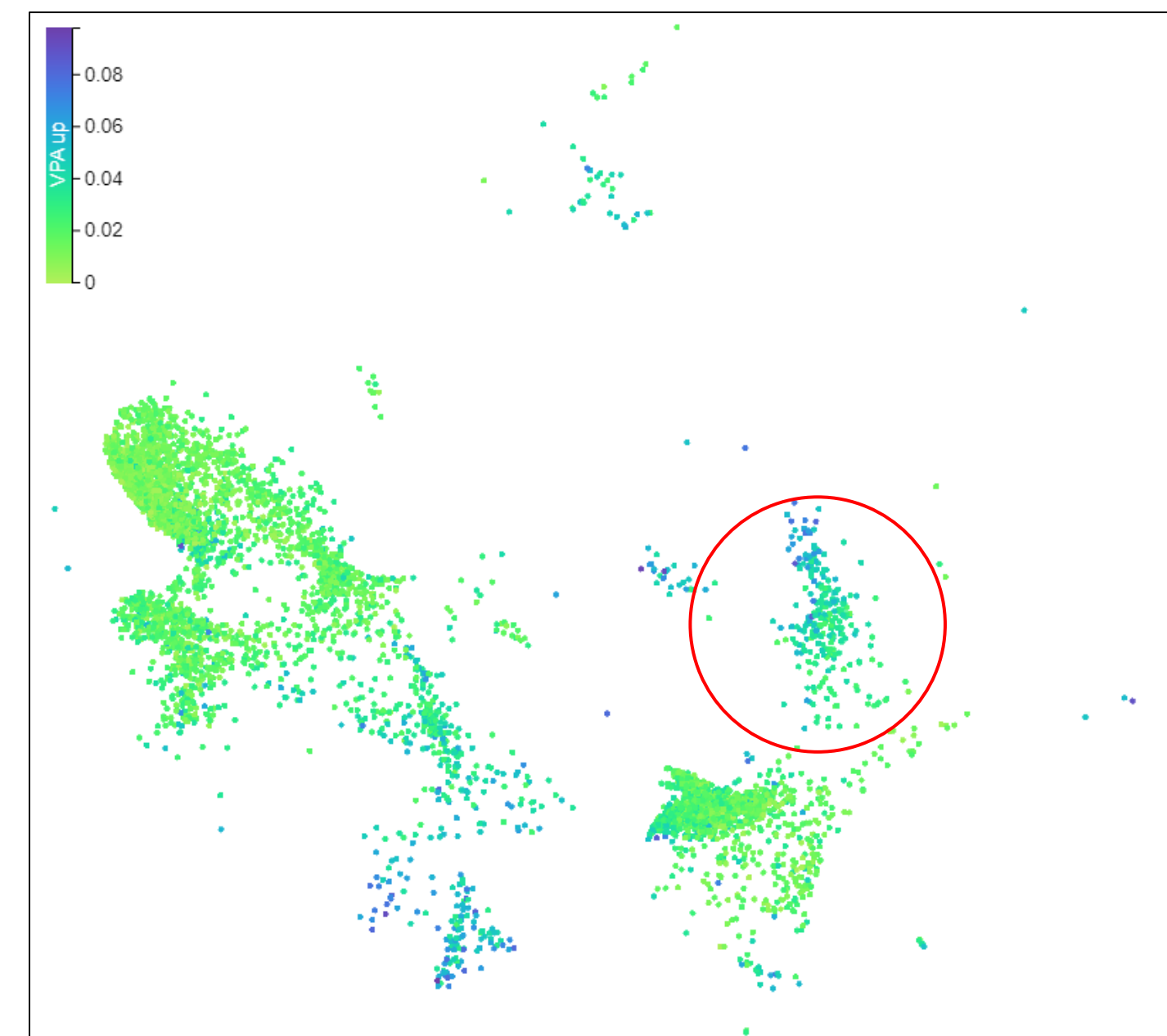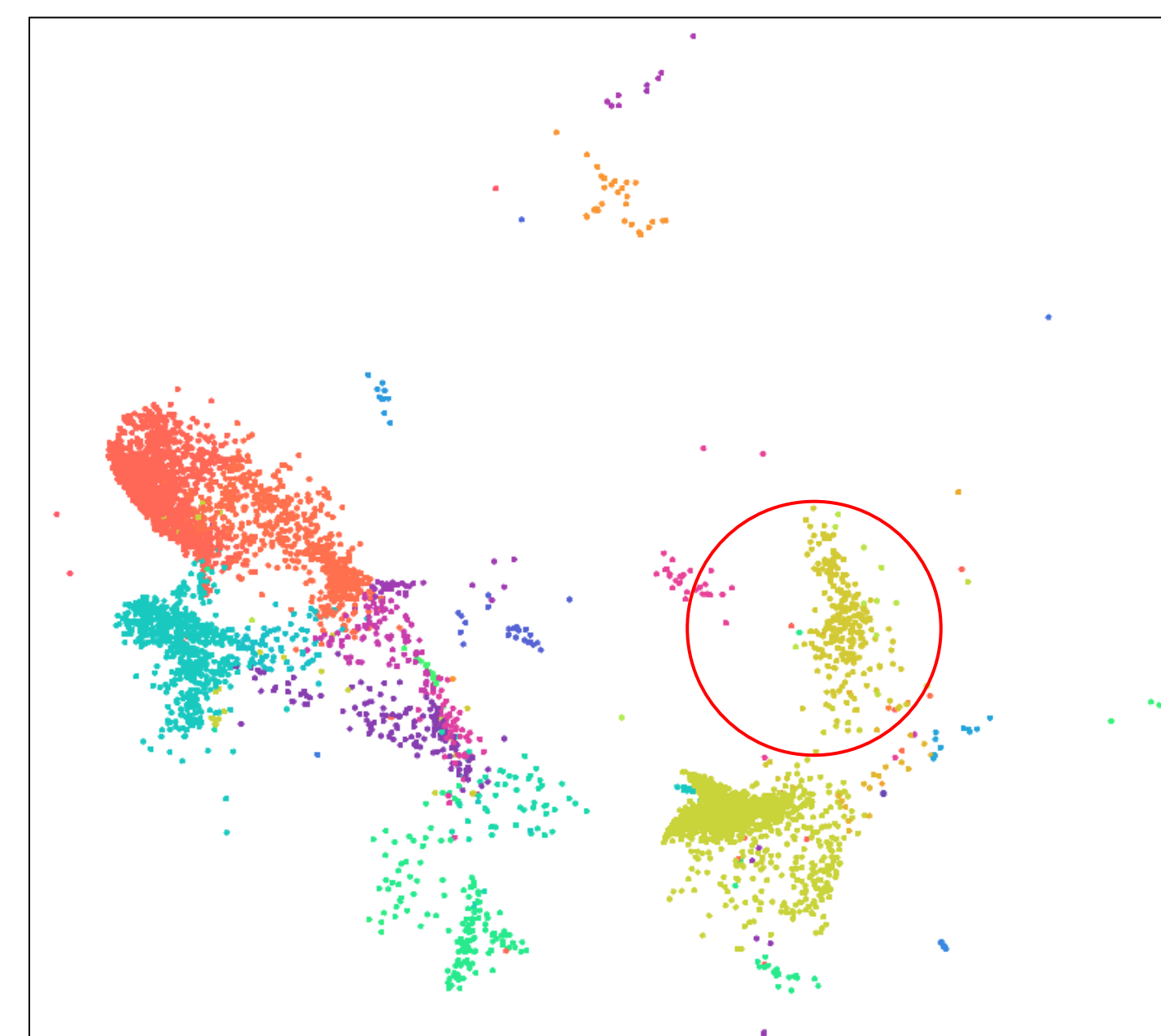**d**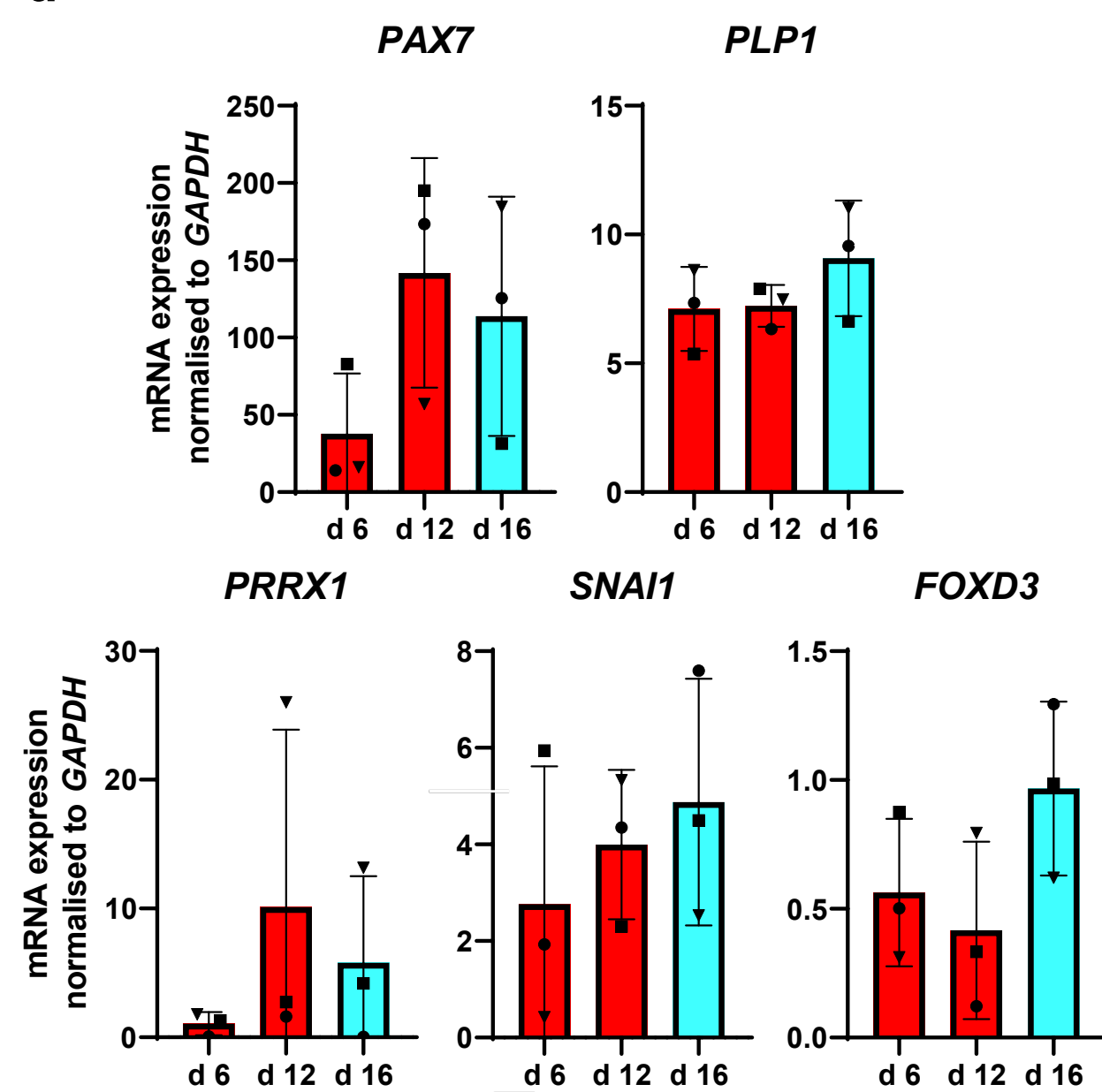**e**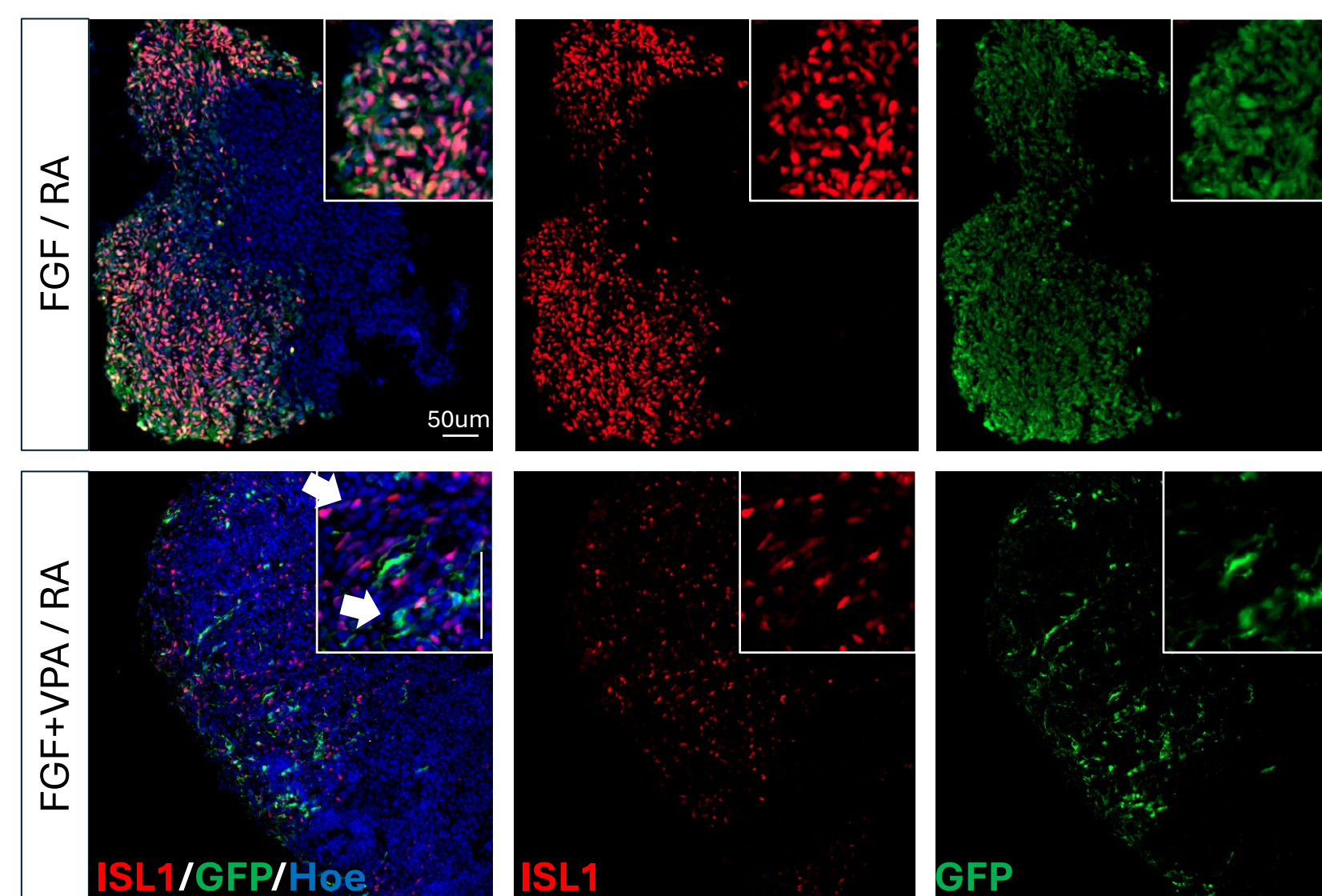**f**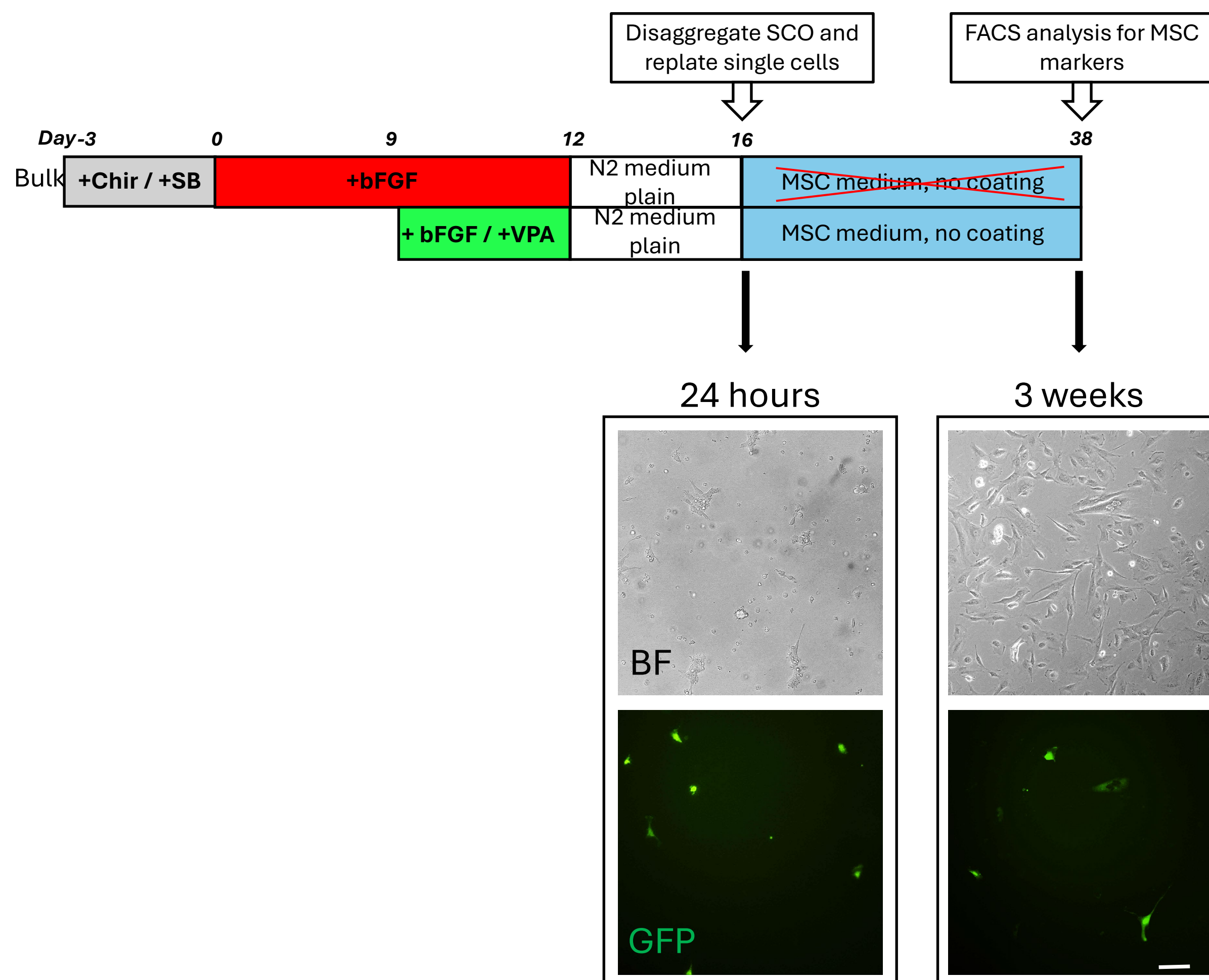**g**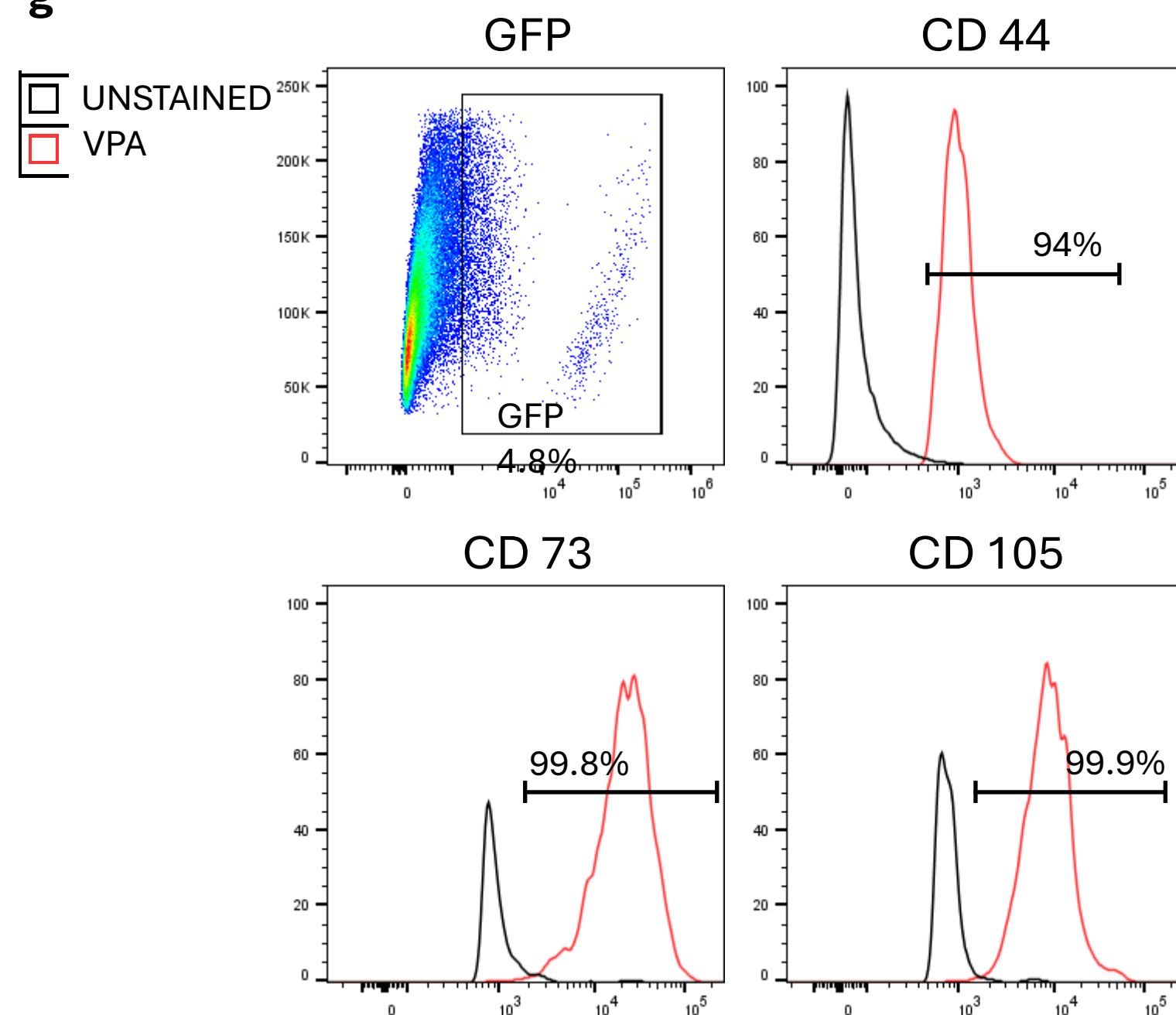

Supplement: Supplementary file 5 — SFig5 [file 41380_2024_2732_MOESM5_ESM.pdf]

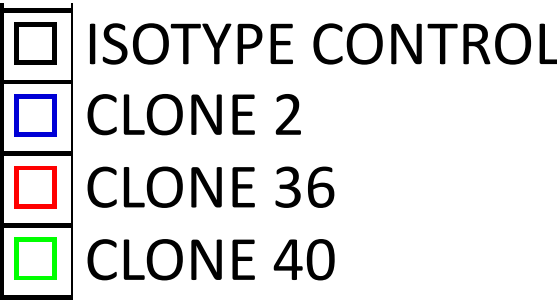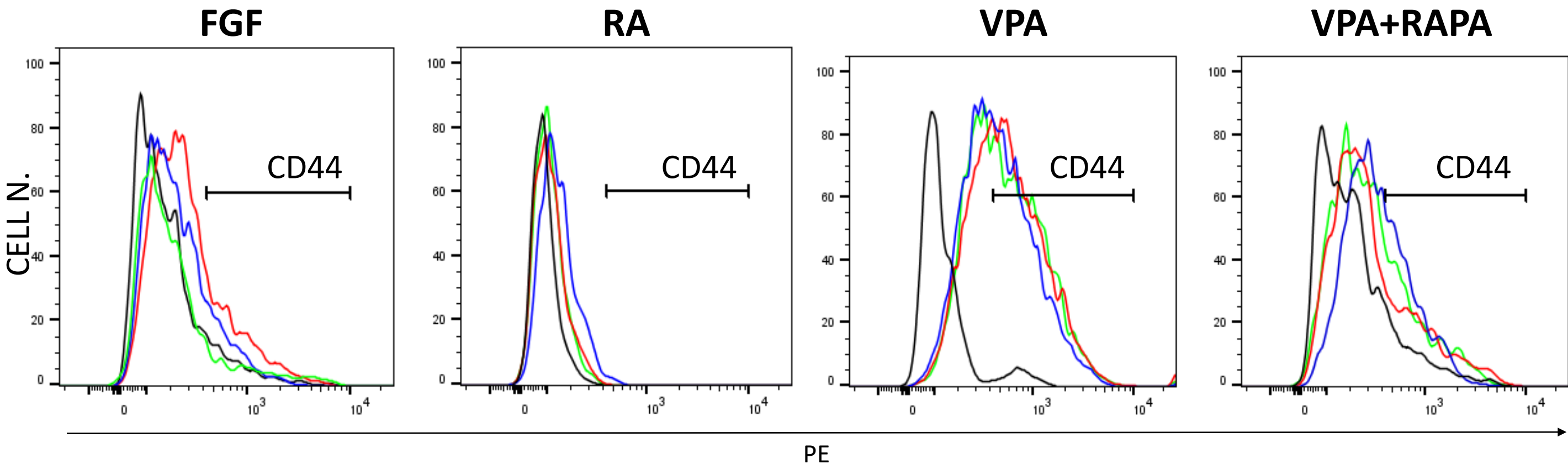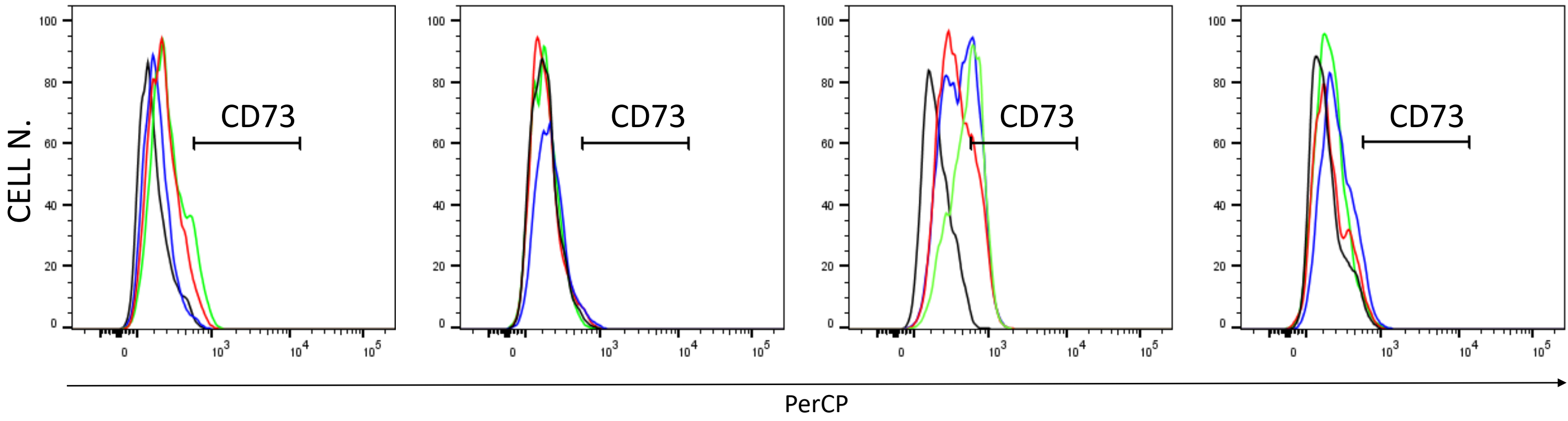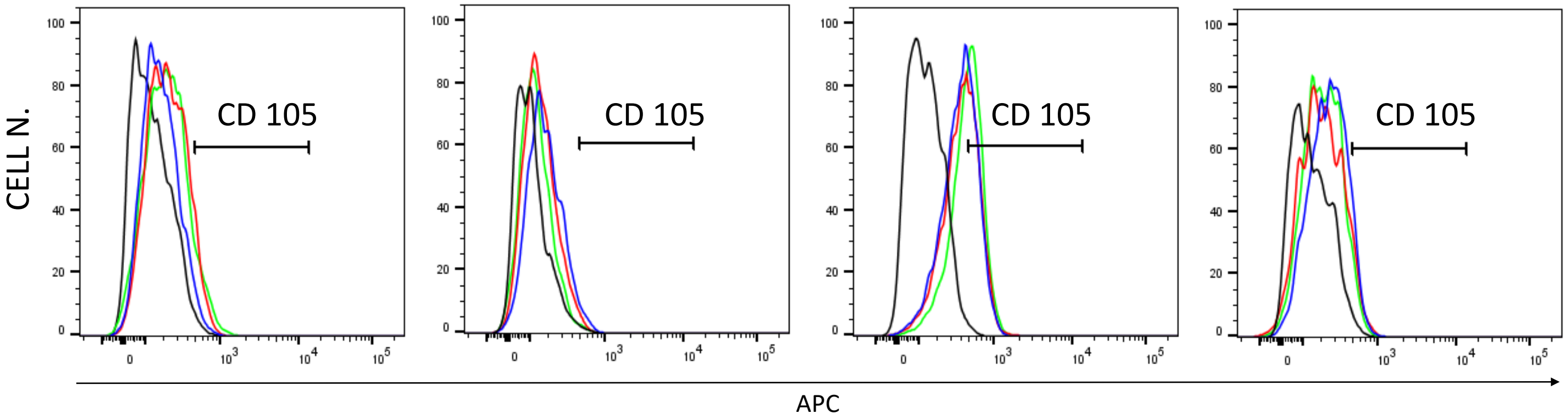

Supplement: Supplementary file 6 — SFig6 [file 41380_2024_2732_MOESM6_ESM.pdf]

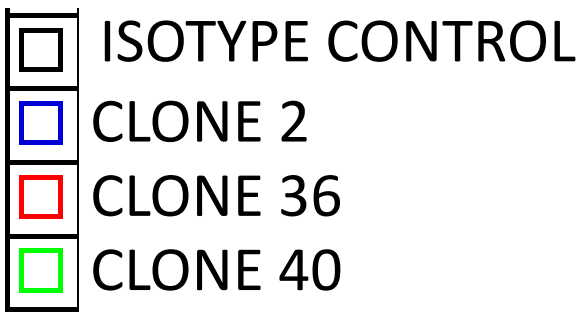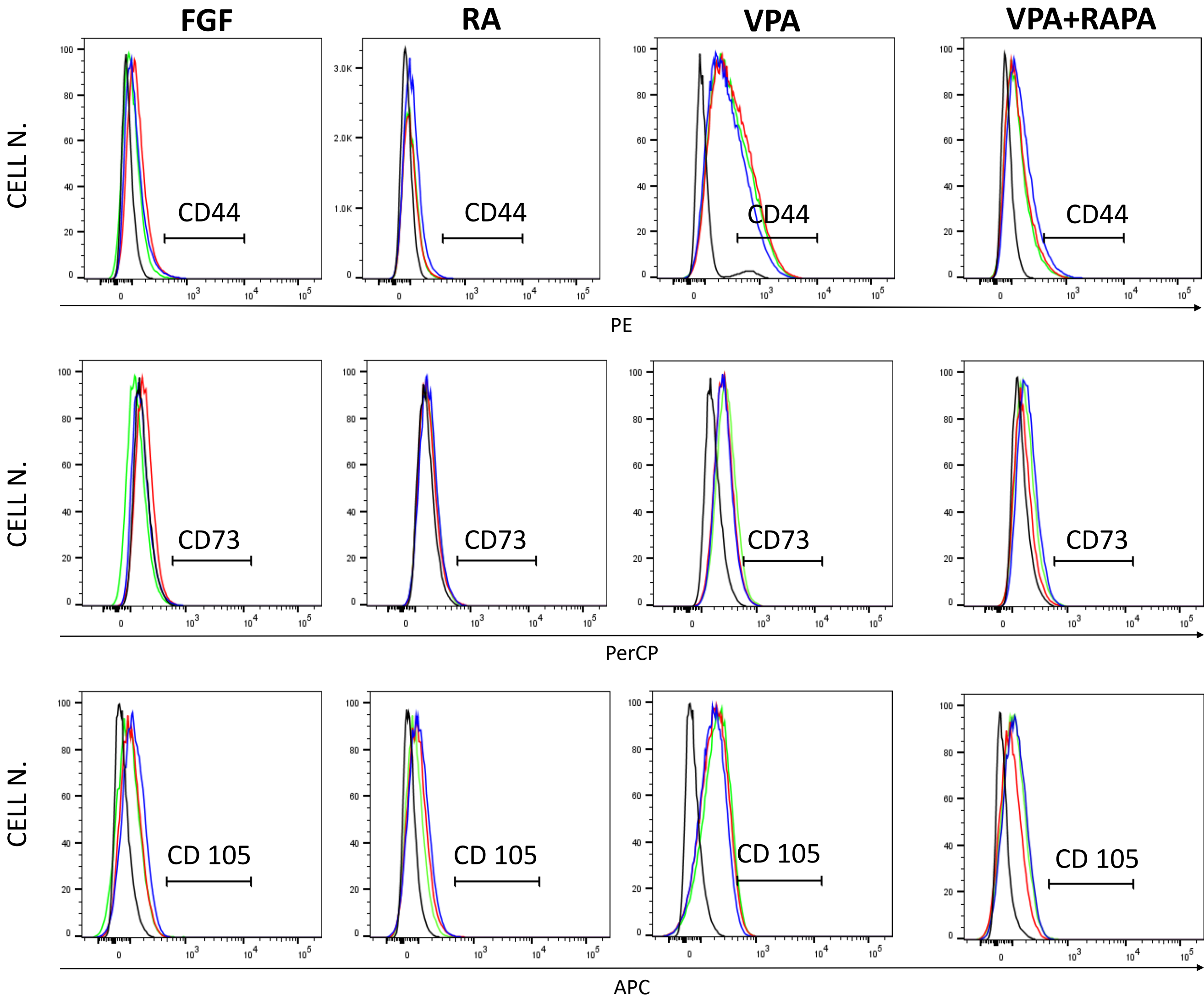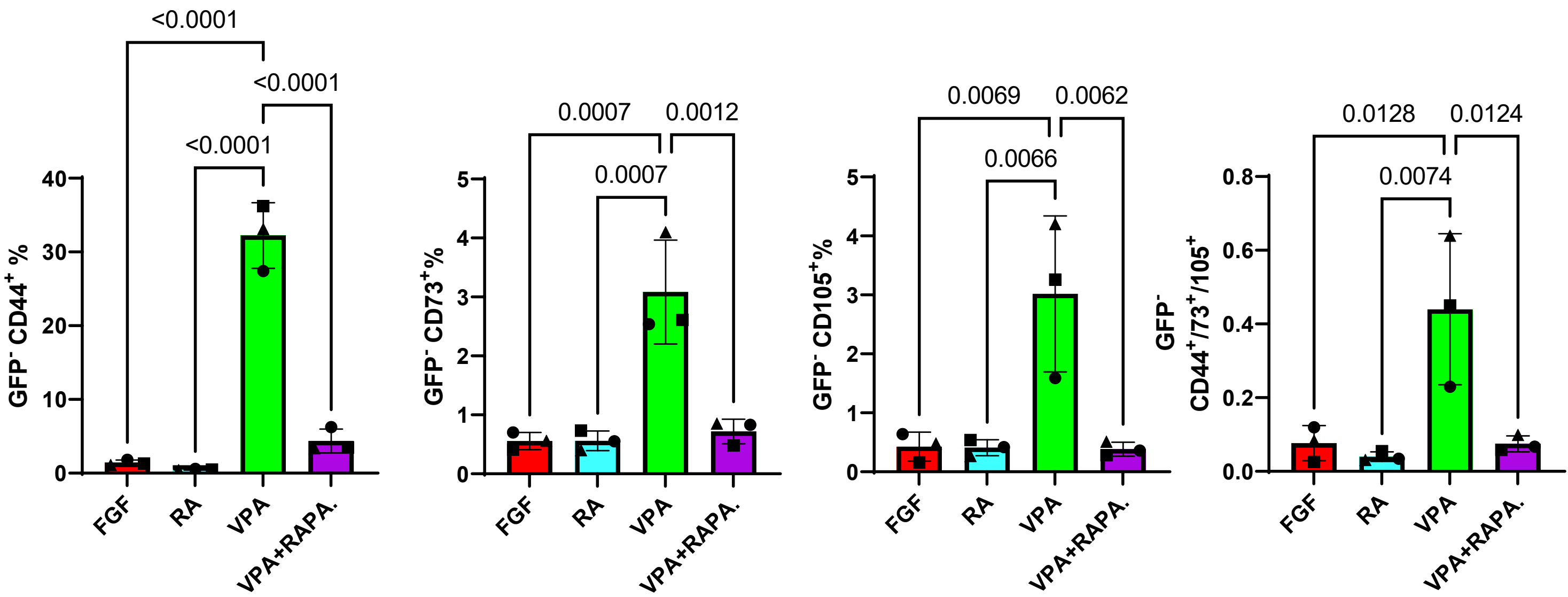

Supplement: Supplementary file 7 — SFig7 [file 41380_2024_2732_MOESM7_ESM.pdf]

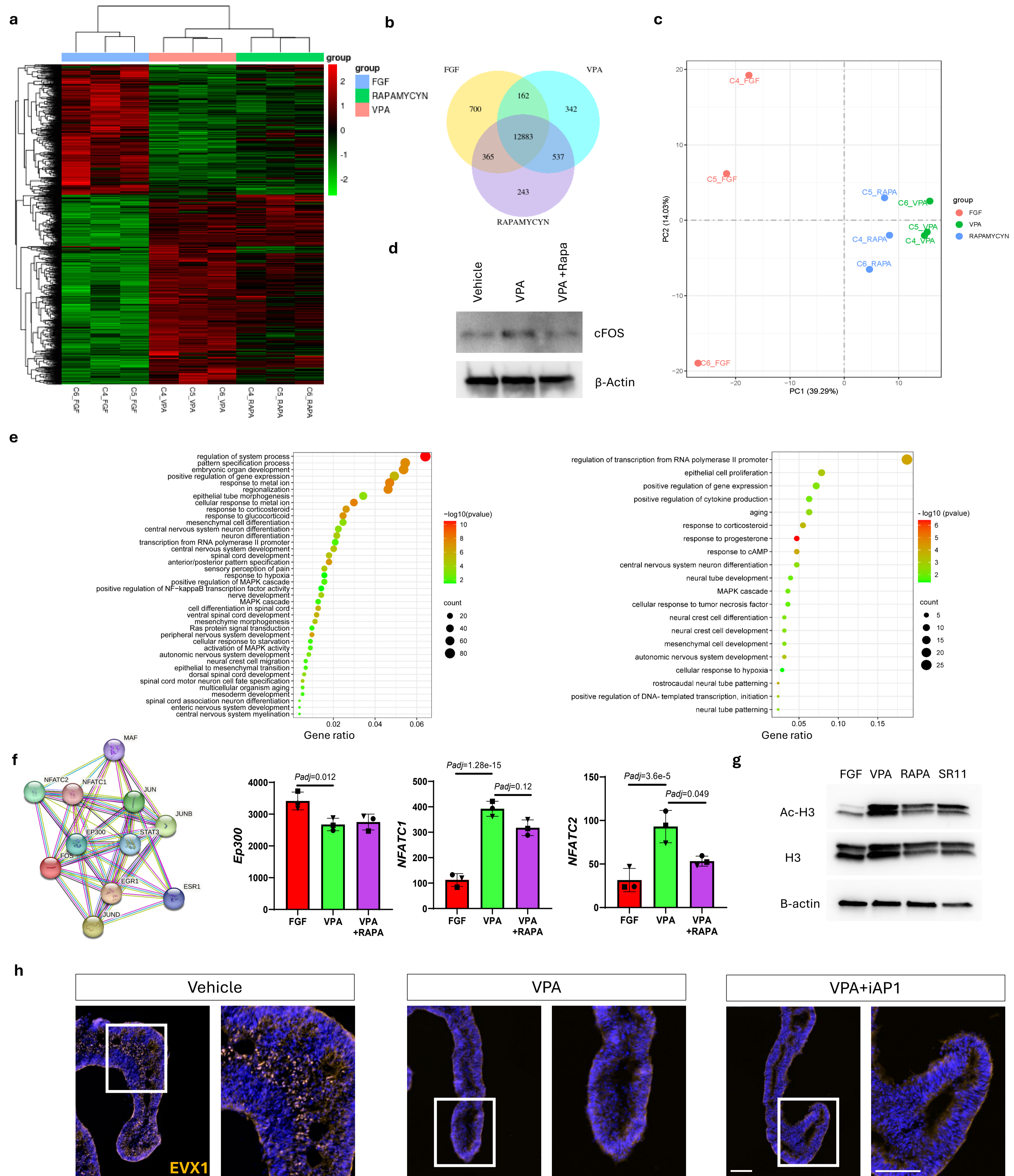

Supplement: Supplementary file 8 — SFig8 [file 41380_2024_2732_MOESM8_ESM.pdf]
