# Supplementary material for: Valproic acid-induced teratogenicity is driven by senescence and prevented by Rapamycin in human spinal cord and animal models
Source: Mol Psychiatry. 2024 Sep 3;30(3):986–98. doi: 10.1038/s41380-024-02732-0 (PMC11835743; doi:10.1038/s41380-024-02732-0)
Supplement: Supplementary file 9 — STable 1 [file 41380_2024_2732_MOESM9_ESM.docx]

|  | Primer |  | Sequence |
| --- | --- | --- | --- |
|  | Li F |  | CAGCAGAGAAGCTGATGAGAGGAAG |
|  | LiR |  | TAGGAATGCTCGTCAAGAAGACAGG |
|  | SeF |  | CAGATGCGACTTCAGAACCA |
|  | Se R |  | ATGTCGAAGCCTCAGGCTGTT |
|  | RiF |  | acaaacgtggtgggcaactgg |
|  | RiR |  | CTTTATTCAAATACCACTGGGAGGC |
|  |  |  |  |
|  |  |  |  |
| Gene | Direction |  | Sequence |
| *SOX10* | Forward |  | CATCCACCTCACAGATCGCC |
|  | Reverse |  | GCATGTCAGACCCTCACTATCTG |
| *FOXD3* | Forward |  | GACGCAGGTTGCGATAGCC |
|  | Reverse |  | CGCCTCCTTGGGCAATGTC |
| *CDH19* | Forward |  | ATCTGCACCCACTGGGACTT |
|  | Reverse |  | CTGCTCAGGAACATGATGG |
| *MPZ* | Forward |  | GGTCCCCCACTTICTCAACC |
|  | Reverse |  | TGTAAACCACGATGGCCTGG |
| *NGFR* | Forward |  | CCTACGGCTACTACCAGGATG |
|  | Reverse |  | CACACGGTGTTCTGCTTGT |
| NEOMYCIN | Forward |  | AGACAATCGGCTGCTCTGAT |
|  | Reverse |  | ATACTTTCTCGGCAGGAGCA |
| GFP | Forward |  | AAGGGCATCGACTTCAAGG |
|  | Reverse |  | TGCTTGTCGGCCATGATATAG |
| mMaple | Forward |  | CAGATGCGACTTCAGAACCA |
|  | Reverse |  | ATGTCGAAGCCTCAGGCTGTT |
| *SOX9* | Forward |  | AGGAAGTCGGTGAAGAACGG |
|  | Reverse |  | CTGGGATTGCCCCGAGTG |
| *HNK1* | Forward |  | GCAGGTTGACGGCAAATCC |
|  | Reverse |  | CCTGGCGTGGTCTACTTCG |
| *ASCL1* | Forward |  | CGCGGCCAACAAGAAGATG |
|  | Reverse |  | CGACGAGTAGGATGAGACCG |
| *NEUROD1* | Forward |  | ATGACCAAATCGTACAGCGAG |
|  | Reverse |  | GTTCATGGCTTCGAGGTCGT |
| *NEUROD4* | Forward |  | GAGAGCTAGTCAACACACCATC |
|  | Reverse |  | GCATCCCATAAGTACCTGGTCTG |
| *DLL1* | Forward |  | GACGAACACTACTACGGAGAGG |
|  | Reverse |  | AGCCAGGGTTGCACACTTT |
| *PHOX2B* | Forward |  | AACCCGATAAGGACCACTTTTG |
|  | Reverse |  | AGAGTTTGTAAGGAACTGCGG |
| *HOXB1* | Forward |  | GAGCTTTGCACCGGCCTAT |
|  | Reverse |  | CTTCATCCAGTCGAAGGTCCG |
| *OCT4* | Forward |  | GTACTCCTCGGTCCCTTTCC |
|  | Reverse |  | CAAAAACCCTGGCACAAACT |
| *NANOG* | Forward |  | CAGCCCTGATTCTTCCACCAGTCCC |
|  | Reverse |  | TGGAAGGTTCCCAGTCGGGTTCACC |
| *SOX2* | Forward |  | TACAGCATGTCCTACTCGCAG |
|  | Reverse |  | GAGGAAGAGGTAACCACAGGG |
| *CD44* | Forward |  | CTGCCGCTTTGCAGGTGTA |
|  | Reverse |  | CATTGTGGGCAAGGTGCTATT |
| *CD90* | Forward |  | ATCGCTCTCCTGCTAACAGTC |
|  | Reverse |  | CTCGTACTGGATGGGTGAACT |
| *CD105* | Forward |  | GCATCCTTCGTGGAGCTACC |
|  | Reverse |  | GAGGAGTGGTCTGGATCGG |
| *SNAIL1* | Forward |  | TCGGAAGCCTAACTACAGCGA |
|  | Reverse |  | AGATGAGCATTGGCAGCGAG |
| *PRRX1* | Forward |  | TGATGCTTTTGTGCGAGAAGA |
|  | Reverse |  | AGGGAAGCGTTTTTATTGGCT |
| *PAX7* | Forward |  | GTCTCCAAGATTCTTTGCCG |
|  | Reverse |  | CCACCTGTCTGGGCTTGCTG |
| *PLP1* | Forward |  | TGCTGATGCCAGAATGTATGG |
|  | Reverse |  | GCAGATGGACAGAAGGTTGGA |
|  |  |  |  |
|  |  |  |  |
|  |  |  |  |
|  |  |  |  |
