# Supplementary material for: Valproic acid-induced teratogenicity is driven by senescence and prevented by Rapamycin in human spinal cord and animal models
Source: Mol Psychiatry. 2024 Sep 3;30(3):986–98. doi: 10.1038/s41380-024-02732-0 (PMC11835743; doi:10.1038/s41380-024-02732-0)
Supplement: Supplementary file 11 — Supplementary Figures legends [file 41380_2024_2732_MOESM11_ESM.docx]

**Supplementary Figures Legends**

**Sfig1**

**a** Sanger sequencing of the 5’ and 3’ ends of the switch integrated in the *SOX10* locus to verify homology driven repair and occurrence of potential indels in bulk targeted population. **b** FACS sorting measuring the activation of the genetic switch expressing GFP over the differentiation protocol from a bulk iPSCs population to SCPs. **c** Representative live image showing co-localization of GFP (green) and photoconverted mMaple (red) in differentiated SCPs. **d** Quantification of GFP, mMaple and SOX10 positive SCPs generated from three clones and fraction of co-localization of relevant markers. **e** Quantification by qPCR of iPSCs markers for pluripotency *OCT4*, *NANOG* and *SOX2* in each clone at the iPCS stage (white) and after differentiation into SCPs (red). Each symbol represents a different clone; two-tailed Student's t-test. error bars represent s.d. **f** Representative field of live fluorescent microscopy of SCPs untreated (CTRL) and after 6 days exposure to Geneticin.

**Sfig2**

**a** Schematic representation of the differentiation protocol of iPSCs into SCPs, further differentiation into MSCs, and specification towards adipocytes and osteoblasts. **b** Representative live image of MSCs generated from bulk iPSC-derived SCPs after 7 days of differentiation protocol. The channels refer to the excitation frequency of GFP (488nm) and mMaple (562nm). The image shows emission before and after photoconversion. **c** Histogram FACS analysis to identify GFP+ population of MSC generated from each clone (red peak) compared to the original iPCS population (light blue peak). The relative percentage of GFP+ cells is indicated in each graph. After 3 weeks, more than 90% of MSCs were GFP positive, consistent with the GFP percentage detected at the initial SCP stage (Fig1e). **d** qPCR quantification in independent MSCs differentiation of SCPs generated from three clones of SCPs markers expression *SOX10* and *FOXD3*, in **e** of MSCs markers *CD44*, *CD90* and *CD105*, in **f** of transgenes associated with the genetic switch mMaple, GFP and Neomycin resistance. **g** Representative image of MSCs (CTRL) differentiated into osteoblasts (OSTEO) and processed for immunofluorescent staining for Osteopontin (OSTP). Graphs are showing relative % of GFP positive cells in maintained MSCs (CTRL) and after differentiation for all three clones, and the quantification of Osteopontin expression in each independent differentiation. Each data point is the average value of three technical replicates per clone. **h** Representative bright filed image of MSCs and osteoblasts stained with Alizarin red and quantification of the staining by absorbance in three replicates for each clone. **i** Representative images of MSCs and MSC-derived Adipocytes immunolabelled for Fatty Acid-Binding Protein 4 (FABP4, red) and quantification of GFP positive cells and FABP4 in maintained MSCs (CTRL) and after differentiation **of** all three clones. Each data point is the average value of three technical replicates per clone. Notably, the undifferentiated MSCs population retained strong GFP expression after 3 weeks from specification. **l** Representative bright field image of MSCs and Adipocytes stained with Oil red and quantification of the staining by absorbance of three technical replicates for each clone. Significance is indicated per each column; Two-tailed Student's t-test, error bars represent s.d.

**SFig3**

**a** 3D projection of a whole mount SCO, GFP+ cells in green, mMaple+ cells in red. **b** Imaging on a tabletop fluorescence microscope of the organoid reconstructed in (a). **c** Region of interest of whole organoid live imaging at the beginning and at the end of the session of 96 hours. Photoconversion identifies two groups of GFP positive cells, one mMaple positive and the other mMaple negative. Photoconversion was renewed every 24 hours to identify newly specified neural crest cells. **d** Dividing mMaple positive single cell tracked over time. **e** Left panel, green indicates the trajectories of GFP only cells, while red indicates GFP/mMaple positive cells. Right panel, trajectories of identified single cells are traced and color-coded. **d** Box plot quantification of speed and ratio of displacement/time for mMaple positive and negative cells (in a.u.), Two-tailed Student's t-test. **e** Quantification of axial displacement on the x (upper panels) and y (lower panel) axis over time of every tracked cell (left panels). Gradient color shows the degree of displacement on the indicated axis for GFP (middle) and mMaple (right panels) cells.

**SFig4**

**a** Representative immunostaining for neuroepithelial marker BRN2 **of** elongating neural tube during proliferation phase. **b** Quantification of the percentage of organoids presenting detectable photoconvertible mMaple signal. Analysis of 3 replicates n= 8-15 organoids. **c** Live imaging of hSCO over the last 4 days of the proliferation phase exposed to vehicle or Valproic acid 2mM. Green shows emission at 520nm, red shows emission at 600nm after UV-induced photoconversion. Bar 50 µm. **d** Quantification by qPCR for the presence of transcripts of the NCC markers *SOX10*, *SOX9* and *HNK1* in samples extracted from 3 independent batches of SCOs generated from bulk iPSC population, n=3-6 SCOs were **pooled** for each sample. Two-tailed Student's t-test, error bars represent s.d. **e** Western blot analysis for SOX10 protein content in SCOs exposed to increasing concentrations of Valproic acid. β-actin was used to control for protein loading. 8-12 organoids were **pooled** for each lysate and 100 µg was loaded per lane. **f** Western blot analysis for the indicated proteins and phosphorylated forms in SCOs exposed to increasing concentrations of Valproic acid. Loading as per **e**.  **g** GO term enrichment analysis showing the Top 8 biological processes emerging from the list of genes differently expressed between VPA and VPA+Rapamycin groups. **h** HSCO were digested by incubation with Accutase, resulting single cells were passed through a mesh strain to remove doublets and analysed by FACS analysis. Plot shows an example of gating used to differentiated GFP negative and positive cells. Bar graph shows the percentage of GFP positive cells present in SCOs exposed to the indicated compounds, in three replicates each with n=4-7 individual organoids pooled. One-way ANOVA, error bars represent s.d. **i** Relative quantification by qPCR for the presence of the indicated markers of neuronal lineage in SCOs exposed to VPA. n=3-6 SCOs were pulled for each sample; two-tailed Student's t-test, error bars represent s.d. **j** Genes significantly downregulated by VPA (*Padj*<0.05) in bulk RNA-sequencing data are highly abundant in the neuron cluster (left panel) as identified by mapping on single cell spinal cord transcriptomic database. Gene set mean expression shown in color scale (right).

**SFig5**

**a** Relative RNA expression of MSCs markers normalized to *GAPDH* mRNA in 4-5 pooled SCOs of 5 independent replicates exposed to vehicle or VPA. Error bars represent s.d.; two-tailed Student's t test, ** P<0.01. **b** Heat map shows selected MSC markers measured via bulk RNAseq across three SCOs independent replicates exposed to vehicle or Valproic Acid. **c** Bulk RNAseq identified genes significantly upregulated by VPA (*Padj*<0.05) mapping onto single cell transcriptome data of 13^th^ week post-fertilization stage human male spinal cord, donor 8. Fetal mesenchymal progenitor cluster is circled in red. Gene set mean expression shown in color scale. **d** Total RNA was extracted from 3 groups of 4-5 pooled SCOs generated from bulk iPSCs at different timepoints during the differentiation and the expression of the indicated MSC markers was examined by qPCR and quantified relative to *GAPDH*; One-way ANOVA, error bars represent s.d. **e** representative immunostaining for ISL1 (red) of SCOs exposed to 4 days of RA after 12 days of FGF or 8 days FGF/4days FGF+VPA, and overlay with GFP (green). White arrows show loss of co-localization between ISL1 staining and GFP. **f** Schematic representation of the experimental plan showing the timeline of exposure to different compounds in SCO medium, followed by medium without additional compounds (N2 medium plain), and plating of single cells on a 6-well plate in MSC medium. Arrows indicate representative bright field images and at 488nm excitation of cells from disaggregated SCOs after the indicated time in culture. Cells derived from the FGF SCO group failed to attach. **g** FACS analysis for the indicated MSC markers of GFP positive cells derived from SCOs exposed to VPA and grown in culture in MSC medium for three weeks.

**SFig6**

Individual FACS analysis of each clone and each marker used for the quantification shown in fig 4f.

**SFig7**

Individual FACS analysis for the expression of MSCs markers of the GFP negative fraction of fig 4f for each clone, and quantification. Gating was kept equal for GFP positive and negative analysis.

**SFig8**

**a** Heatmap and unsupervised clustering of the whole transcriptome by bulk RNA sequencing of the indicated samples. **b** Venn diagram showing differentially expressed transcripts between SCOs exposed to vehicle (FGF), VPA and VPA+Rapamycin (RAPAMYCIN) with a significance *AdjP* value <0.05. **c** Principal component analysis of the three replicates of each condition used during bulk RNA sequencing and subsequent analysis. **d** Western blot analysis of protein samples extracted from SCOs exposed to the indicated compounds for the expression of cFOS and loading control shown by β-actin. **e** GO term enrichment analysis of differentially expressed genes identified as significantly differently expressed between Vehicle and VPA groups (left) and between VPA and VPA+Rapamycin groups (right). **f** Network of genes closely associated by interaction at multiple levels to FOS and genes belonging to the FOS network showing significantly differential expression after the indicated treatments. The number of readings was extracted from the bulk RNA sequencing data. **g** Western blot analysis for the content of acetylated H3 (Ac-H3), histone H3 and β-actin in protein samples extracted from SCOs exposed to the indicated compounds. **h** Representative field of view of sections of SCO exposed to vehicle, VPA, and VPA+iAP1 and immunolabeled for the interneuronal marker EVX1 (orange).

**STable1**

List of primers used to verify genome editing and for qPCR expression analysis.

**STable2**

Senescence-associated genes were identified using the HAGR database on microarray transcriptomic profiling of folding neural tube organoids exposed to VPA.
